# A single-agent fusion of human IL-2 and anti-IL-2 antibody that selectively expands regulatory T cells

**DOI:** 10.1038/s42003-024-05987-z

**Published:** 2024-03-09

**Authors:** Yuan Lin, Xue Wang, Yuhao Qin, Chengpan Wang, Tang Zhou, Long Zhang, Lu Su, Wenming Ren, Cheng Liao

**Affiliations:** 1 Shanghai Shengdi Pharmaceutical Co. Ltd, Shanghai, 200100 China; 2 Jiangsu Hengrui Pharmaceutical Co. Ltd, Lianyungang, 222000 China

**Keywords:** Recombinant protein therapy, Systemic lupus erythematosus

## Abstract

The occurrence of many autoimmune diseases takes root on the disrupted balance among Treg cells, Teff cells, etc. Low-dose interleukin-2 (IL-2) cytokine demonstrates promising clinical efficacy in the expansion of Treg cells and the treatment of autoimmune diseases. However, its clinical application is hindered by the small therapeutic index and short half-life. Previous studies have shown that non-covalent complex of human IL-2 and anti-IL-2 antibody biases cytokine activity towards Treg cells and extends IL-2’s half-life. The clinical translation of such complex is non-trivial. In this study, we discover an anti-human IL-2 antibody and engineer a covalently-linked single-agent fusion of human IL-2 and its antibody that selectively expands Treg cells and exhibits superior disease control activity in animal models of ulcerative colitis and systemic lupus erythematosus, with proper safety profile and good developability. These studies pave the road for its clinical development in diverse autoimmune diseases.

## Abstract (web summary)

A single-agent fusion of human IL-2 and antiIL-2 antibody is discovered and engineered to selectively expand regulatory T cells and to treat autoimmune diseases.

## Introduction

Interleukin-2 (IL-2) is a pleiotropic type-I cytokine that is critical for immune homeostasis. It signals via either the trimeric high-affinity receptor composed of IL-2Rα (CD25), IL-2Rβ (CD122) and IL-2Rγ (CD132) or the dimeric intermediate-affinity receptor composed of IL-2Rβ and IL-2Rγ. The trimeric receptor is constitutively and abundantly expressed on regulatory T (Treg) cells, whereas the dimeric receptor is mostly expressed on effector immune cells, such as NK and naïve CD8^+^ T cells. As a result, low-dose IL-2 is sufficient to activate Treg cells, whereas naïve effector cells are only responsive to high-dose IL-2. Upon the formation of IL-2/receptor complex, the IL-2-induced heterodimerization of IL-2Rβ and IL-2Rγ leads to the activation of intracellular JAK/STAT-5 pathway and the proliferation of the responding immune cells.

IL-2 plays a key role in immune tolerance to naturally occurring self-antigens by maintenance of Treg cells. Genetic deletion of IL-2 or IL-2Rs in mice can be lethal, as a result of severe self-attack^1–3^. Extensive preclinical studies demonstrated the effectiveness of low-dose IL-2 in treating autoimmune diseases^4,5^. Recently, low-dose IL-2 has been clinically verified to ameliorate numerous autoimmune diseases, including type 1 diabetes, chronic graft versus host diseases and systemic lupus erythematosus (SLE)^6–8^. However, the clinical application of low-dose IL-2 has been hindered by the requirement of careful and frequent dosing due to the small therapeutic index and short half-life of IL-2.

Multiple approaches have been pursued to overcome the therapeutic limitation of IL-2 in treating autoimmune diseases. One approach is to discover anti-IL-2 antibodies that modulate IL-2 to preferentially bind to the high-affinity receptor IL-2Rαβγ over the intermediate-affinity receptor IL-2Rβγ. For example, the non-covalent complex of mouse IL-2 with an anti-mouse IL-2 antibody JES6-1 considerably expands Treg cells but barely activates NK and naïve CD8^+^ T cells^9^. JES6-1 exerts its function through sterically blocking the IL-2/IL-2Rβ and IL-2/IL-2Rγ interactions, but also allosterically dampening the IL-2/ IL-2Rα interaction^10^. JES6-1 also results in the prolongation of IL-2’s half-life, which is dependent on neonatal Fc receptors (FcRn) and is indispensable to the activity of the IL-2/JES6-1 complex^11^. Subsequent animal studies have demonstrated that IL-2/JES6-1 complex can prevent the development of many autoimmune diseases^12–16^. The proof-of-concept Treg-biasing antibody against mouse IL-2 has triggered a campaign of discovering its counterparts against human IL-2 with potential clinical application in human autoimmune diseases. Indeed, several anti-human IL-2 antibodies have been discovered later to bias the activity of human IL-2 to Treg cells through diverse structural mechanisms^17,18^.

Nonetheless, the drug development and clinical administration of the non-covalent complex of IL-2 and anti-IL-2 antibody is practically challenging. The complex formation, depending on a series of ionic, hydrophobic and Van Der Waals interactions, may be significantly influenced by drug formulation. The balance between antibody-bound and unbound cytokine may be altered dramatically upon the drug administration as the complex concentration decreases in vivo over time. In order to make a more stable molecule with defined ratio between IL-2 and anti-IL2 antibody, mouse IL-2 has been previously covalently linked to anti-mouse IL-2 antibodies, including JES6-1, through a peptide linker^19^. The intramolecular interaction greatly enhances the apparent affinity between IL-2 and JES6-1 to such a degree that the antibody cannot be exchanged by the trimeric IL-2R and that mutations modulating antibody/cytokine affinity have to be made to recapitulate the Treg bias elicited by non-covalent IL-2/JES6-1 complex^19^. These studies demonstrated as proof of concept that a covalently-modified IL-2 by anti-IL-2 antibody is possible, at least for mouse IL-2.

Here we describe the development of a covalently-linked human IL-2/anti-human IL-2 antibody complex as a single-agent fusion protein that expands Treg cells selectively both in vitro and in vivo. We demonstrated that the human IL-2 covalently modified by anti-human IL-2 antibody suppresses the cellular and humoral immunity and controls the disease progression in animal models of ulcerative colitis (UC) and SLE. The IL-2/antibody fusion we discovered can potentially be used in the treatment of a broad spectrum of autoimmune diseases in human.

## Results

### Discovery and in vitro characterization of anti-human IL-2 antibody

**Fig. 1:**
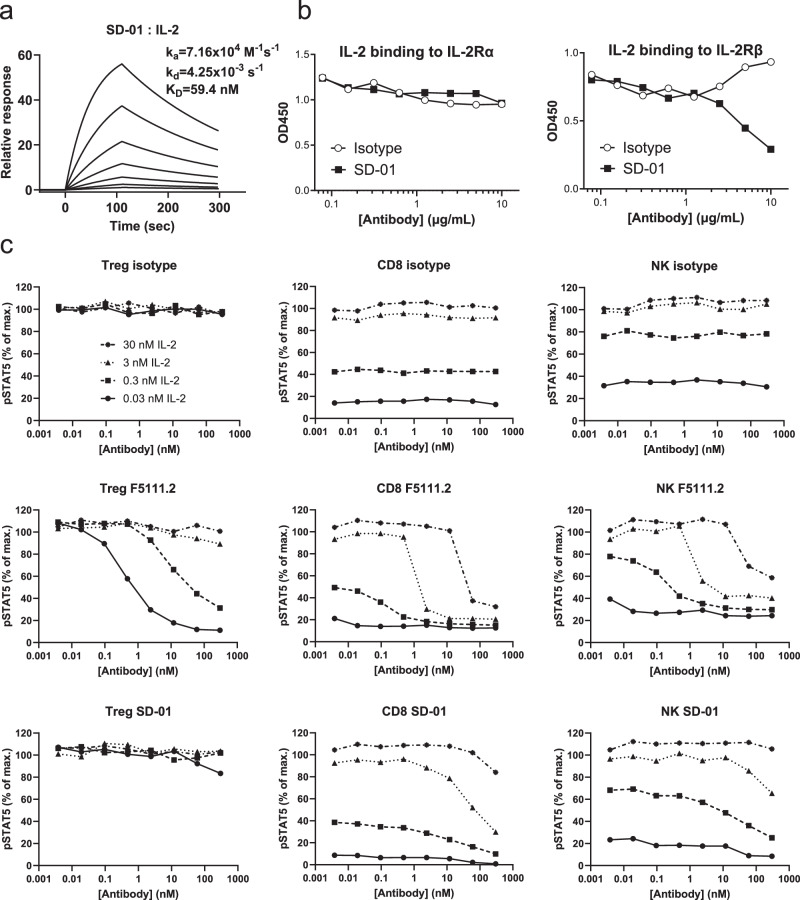
In vitro characterization of anti-human IL-2 antibody, SD-01. **a** SPR kinetic binding analysis of the antibody-human IL-2 interaction. **b** ELISA measurement of the blockage of biotinylated IL-2 binding to plate-coated IL-2Rα or IL-2Rβ by SD-01. Isotype, open circle; SD-01, closed square. **c** STAT5 phosphorylation responses of human Treg, CD8^+^ T and NK cells to serial dilutions of antibody (isotype, SD-01 or F5111.2) in complex with four different concentrations of human IL-2. 0.03 nM IL-2, circle and solid line; 0.3 nM IL-2, square; 3 nM IL-2, triangle; 30 nM circle and dash-dotted line.

Anti-human IL-2 antibodies were identified using a naïve Fab phage library derived from human peripheral blood mononuclear cell (PBMC). One clone, named as SD-01, was developed into a full-length immunoglobulin G (IgG). Purified SD-01 bound IL-2 with an affinity of 59.4 nM characterized by surface plasmon resonance (SPR) (Fig. 1a). Partly due to the relatively low affinity for IL-2, SD-01 hardly inhibited IL-2 binding to IL-2Rα but blocked IL-2 binding to IL-2Rβ only at high concentrations in an enzyme-linked immunosorbent assay (ELISA) (Fig. 1b). To investigate how the receptor blockade activity is translated to cellular activity, SD-01 was further assessed for its ability to modulate the activity of IL-2 to stimulate pSTAT5 signaling in different cell subsets of human PBMC. Treg, CD8^+^ T and NK cells were divided on the basis of the expression of CD3, CD4, CD8, CD25, CD56 and Foxp3 (Supplementary Fig. 1). IL-2 at a concentration as low as 0.03 nM was able to induce maximal pSTAT5 signaling in Treg cells, whereas at least 100-fold or higher concentrations of IL-2 were needed to achieve comparable STAT5 activation in CD8^+^ T and NK cells (Fig. 1c), consistent with previous report^18,20^. We then stimulated human PBMC with serial dilutions of SD-01 in non-covalent complex with different concentrations of human IL-2. SD-01 had little impact on IL-2-induced phosphorylation of STAT5 in Treg cells, whereas IL-2/STAT5 signaling was strongly inhibited in CD8^+^ T and NK cells (Fig. 1c), suggesting that SD-01 further enhanced the biased activity of IL-2 towards Treg cells. In comparison, F5111.2, an antibody previously reported to be a strong IL-2 binder with sub-nanomolar affinity, inhibited pSTAT5 signaling not only in CD8^+^ T and NK cells but also in Treg cells, suggesting that overdose of F5111.2 may be detrimental for IL-2-induced proliferation of Treg cells (Fig. 1c). In conclusion, SD-01 is an anti-IL-2 antibody whose impact on IL-2 activity is more on Treg cells than on CD8^+^ T and NK cells.

### Generation and in vitro characterization of covalently-linked human IL-2/antibody complex

**Fig. 2:**
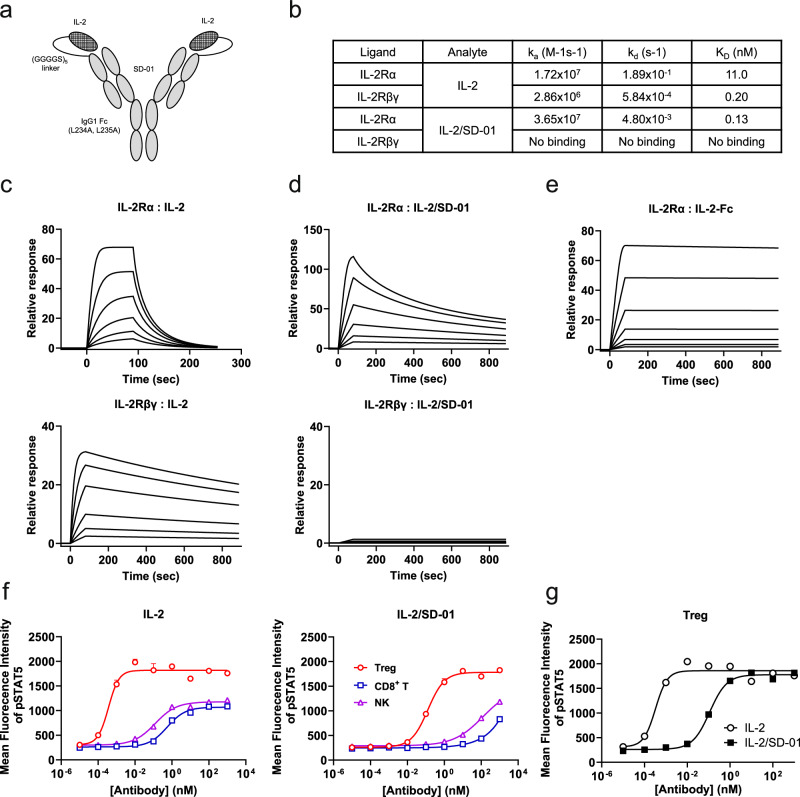
IL-2/SD-01 selectively expands Treg cells in vitro. **a** Schematic of the human IL-2/SD-01 single-agent fusion with the C terminus of human IL-2 linked to the N terminus of the light chain of anti-IL-2 antibody SD-01 via a (Gly_4_Ser)_5_ flexible linker. Its Fc is from IgG1 and contains the mutations of L234A and L235A to remove the interactions with FcγRs. **b** SPR binding kinetics of the interactions between human IL-2 or IL-2/SD-01 with IL-2Rα or IL-2Rβγ. IL-2Rα or IL-2Rβγ was captured on the chip as ligand. IL-2 or IL-2/SD-01 was injected as analyte. The sensorgram of human IL-2 (**c**), IL-2/SD-01 (**d**) or Fc-tagged IL-2 (**e**) binding to IL-2Rα or IL-2Rβγ. **f** STAT5 phosphorylation responses of the indicated human cell subsets to serial dilutions of IL-2 or IL-2/SD-01 fusion. Treg cells, red open circle; CD8 + T cells, blue open square; NK cells, purple open triangle. **g** STAT5 phosphorylation responses of Treg cells to serial dilutions of IL-2 (open circle) or IL-2/SD-01 fusion (closed square).

Compared to previously reported Treg-biasing anti-IL-2 antibodies, such as F5111.2 and UFKA-20, SD-01 has a relatively lower affinity towards IL-2, which may allow the construction of a Treg-biasing covalently-linked IL-2/antibody complex^17,18^. A strong interaction between IL-2 and the antibody may impede the engagement of trimeric IL-2Rs on Treg cells. We generated a series of such covalent complexes by fusing human IL-2 to the N terminus of either the heavy or light chain of anti-IL-2 antibodies via a variable sized linker (i.e., (G_4_S)_3_, (G_4_S)_4_ and (G_4_S)_5_), among which we chose the one with IL-2 fused to N terminus of light chain of SD-01 via (G_4_S)_5_ linker (covalently-linked IL-2/SD-01 complex, or briefly, IL-2/SD-01) for further characterization (Fig. 2a). The Fc part contains the effector-less mutations of L234A and L235A (following Eu numbering scheme) to eliminate possible interactions with FcγR-expressing cells.

We first measured the binding affinity of IL-2/SD-01 towards IL-2Rα and IL-2Rβγ by SPR. As expected, the tag-free IL-2 readily bound to surface-captured IL-2Rα (11.0 nM) and IL-2Rβγ (0.20 nM) (Fig. 2b, c). However, IL-2/SD-01, in which the IL-2 cytokines are sequestered by intramolecular Fab arms of SD-01, has around 85-fold increase in apparent affinity to IL-2Rα (0.13 nM), due to the bivalent nature of IL-2 within IL-2/SD-01 (Fig. 2b, d). In a head-to-head comparison, an Fc-tagged bivalent IL-2 bound IL-2Rα with much slower dissociation kinetics than that of IL-2/SD-01, suggesting that SD-01 can partially interfere with the interaction between IL-2 and IL-2Rα (Fig. 2e). The interference may be so weak that it is only revealed in the context of IL-2/SD-01 fusion protein. On the other hand, IL-2/SD-01, albeit containing bivalent IL-2, was unable to bind IL-2Rβγ at all tested concentrations, indicating that SD-01 fully blocks the interaction between IL-2 binding to IL-2Rβγ (Fig. 2d).

To test the activity of IL-2/SD-01 to cells bearing high- and intermediate-affinity IL-2R, we measured the pSTAT5 level in different cell subsets of human PBMC stimulated with serial dilutions of IL-2 or IL-2/SD-01. As expected, IL-2 stimulated Treg cells (EC50 = 0.0003 nM) at low concentration and CD8^+^ T (EC50 = 0.51 nM) and NK cells (EC50 = 0.11 nM) at intermediate concentration (Fig. 2f). In contrast, IL-2/SD-01 did not activate CD8^+^ T and NK cells at intermediate concentration and also bore a decreased activity towards Treg cells (EC50 = 0.11 nM) in comparison to IL-2, in agreement with its inability to bind IL-2Rβγ (Fig. 2f,g). The increased apparent affinity of IL-2/SD-01 to IL-2Rα compensated the loss of IL-2Rβγ binding and allowed the activation of Treg cells by IL-2/SD-01 at intermediate concentration. Only at high concentration of 1 μM, CD8^+^ T and NK cells were slightly activated by IL-2/SD-01, presumably due to the presence of trace amount of SD-01-unbound IL-2. Overall, IL-2/SD-01 has increased binding to IL-2Rα and diminished binding to IL-2Rβγ and retained the biased activation of Treg cells over CD8^+^ T and NK cells in vitro.

### IL-2/SD-01 extended half-life of IL-2 and selectively activated Treg cells in mice

**Fig. 3:**
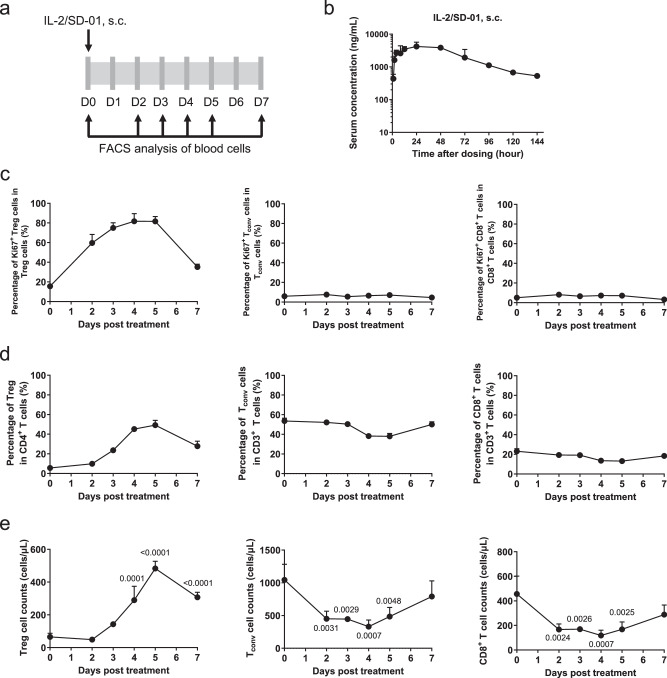
IL-2/SD-01 extends IL-2’s half-life and selectively expands Treg cells in mice. **a** Schematic of pharmacokinetics and pharmacodynamics studies of IL-2/SD-01 in mice. A group of mice (*n* = 3 animals) was subcutaneously injected with a single dose of 0.7 mg/kg IL-2/SD-01. **b** Serum was collected at the indicated time points and the serum level of IL-2/SD-01 was determined by ELISA. Data represent means ± SD. *n* = 3 animals. **c**–**e** Blood was collected at the indicated time points after the administration for flow cytometry analysis. Cells were stained with antibodies against CD3, CD4, CD8, CD25, FoxP3 and Ki67, and gated on Treg, T_conv_ and CD8^+^ T cells. The percentages of Ki67^+^ cells within Treg, T_conv_ and CD8^+^ T cells are shown (**c**). The percentages of Treg within CD4^+^ T cells, T_conv_ cells within CD3^+^ T cells and CD8^+^ T cells within CD3^+^ T cells are also shown (**d**). The total cell counts of Treg, T_conv_ and CD8^+^ T cells are shown (**e**). Data are means ± SD. *n* = 3 animals. Statistical significance of the differences in cell subsets between day 0 and days after the administration of IL-2/SD-01 was determined by one-way ANOVA with Dunnett’s multiple comparisons and the *P* values indicating statistical significance were shown.

To test whether IL-2/SD-01 preferentially activates Treg cells in vivo, Balb/c mice were administered with a single dose of 0.7 mg/kg IL-2/SD-01 subcutaneously (Fig. 3a). In contrast to the short serum half-life (less than 15 min) of IL-2^21^, IL-2/SD-01 showed much more robust pharmacokinetics with an extended half-life of ~36.9 h (Fig. 3b). Thus, the fusion to SD-01 antibody can prolong the serum half-life of IL-2. We next collected the blood and quantified the abundance and proliferation status of Treg and CD8^+^ T cells pre- and post-treatment of IL-2/SD-01. A single dose of IL-2/SD-01 greatly promoted the expression of Ki67, a cell proliferation marker, in Treg cells, lasting for at least 7 days (Fig. 3c). Accordingly, it increased the percentage of Treg cells within CD4^+^ T cells and its total cell counts by ~ 9 and ~7 folds, respectively, reaching peak on day 5 and maintaining Treg level above baseline until day 7 or longer (Fig. 3d,e). In contrast, the expression of Ki67 within CD4^+^CD25^−^ T_conv_ and CD8^+^ T cells and the relative percentage of T_conv_ and CD8^+^ T cells within CD3^+^ T cells were hardly altered (Fig. 3c, d). Their total cell counts even decreased slightly between day 2 and 5 and returned to baseline at day 7 (Fig. 3e). Overall, IL-2/SD-01 preserves the biased activation towards Treg cells over CD8^+^ T cells in vivo and the activation is more persistent partly due to its longer serum half-life.

### IL-2/SD-01 was immuno-suppressive by inducing Treg cells in vivo

**Fig. 4:**
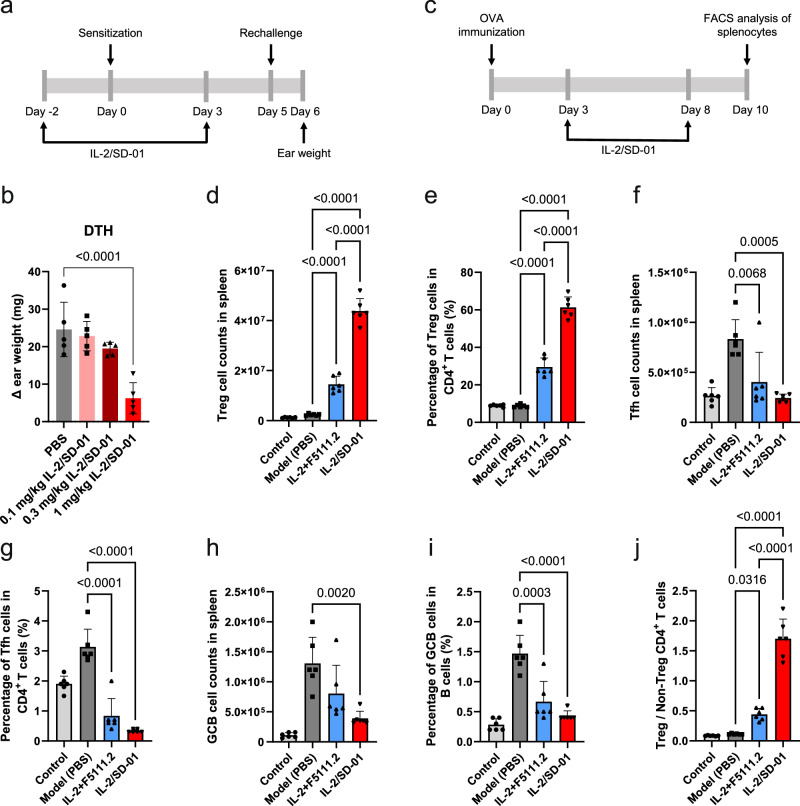
IL-2/SD-01 is immunosuppressive in vivo. **a** Schematic of the experimental design of the mouse delayed-type hypersensitivity study. Mice were sensitized on day 0 through topical application of solution of 2,4-dinitrofluorobenzene (DNFB) to the shaved abdomen. Challenge was performed on day 5 through application of DNFB to the inner and outer surfaces of the right ear. Thickness and weight of both left and right ears were measured on day 6. IL-2/SD-01 was administered subcutaneously on day −2 and 3. **b** The difference of weight between the left and right ears in response to different treatments. Means ± SD and individual values are plotted. *n* = 5 animals. Statistical significance of the differences between PBS-treated group and the IL-2/SD-01-treated groups was determined by one-way ANOVA with Dunnett’s multiple comparisons. PBS, gray bar and closed circle; 0.1 mg/kg IL-2/SD-01, light red bar and closed square; 0.3 mg/kg IL-2/SD-01, dark red bar and closed upward triangle; 1 mg/kg IL-2/SD-01, red bar and closed downward triangle. **c** Schematic of the experimental design of the mouse ovalbumin (OVA) immunization study. Mice were immunized with OVA on day 0 and treated with PBS, IL-2 + F5111.2, or IL-2/SD-01 on day 3 and 8 subcutaneously. The mice in control group was not immunized with OVA and served as negative control. Splenocytes were collected on day 10. The percentages of Treg, Tfh and Germinal Center B (GCB) cells were quantified by FACS. The total cell counts of Treg (**d**) and the percentage of Treg cells within CD4^+^ T cells (**e**) in the spleens of OVA-immunized mice treated with PBS, IL-2 + F5111.2 or IL-2/SD-01 are shown. The total cell counts of Tfh cells (**f**) and the percentage of Tfh cells within CD4^+^ T cells (**g**) in the spleens of OVA-immunized mice treated with PBS, IL-2 + F5111.2 or IL-2/SD-01 are shown. The total cell counts of germinal center B cells (**h**) and the percentage of germinal center B cells within B cells (**i**) in the spleens of OVA-immunized mice treated with PBS, IL-2 + F5111.2 or IL-2/SD-01 are shown. **j** The ratios of cell counts between Treg and non-Treg CD4^+^ T cells are shown. Means ± SD and individual values are plotted. *n* = 6 animals. The *P* values between the immunized groups were determined by one-way ANOVA with Tukey’s multiple comparisons. The control group was not taken into the statistical analysis. PBS, dark gray bar and closed square; IL-2 + F5111.2, blue bar and closed upward triangle; IL-2/SD-01, red bar and closed downward triangle.

The endogenous Treg cells are supposed to be immuno-suppressive. To test whether the Treg cells elicited by IL-2/SD-01 retain the suppressive capacity, mice sensitized and re-challenged with 2,4-dinitrofluorobenzene (DNFB) to induce delayed-type hypersensitivity (DTH), which mainly involves T cells rather than antibodies, were treated with IL-2/SD-01 (Fig. 4a). Compared with PBS, IL-2/SD-01 suppressed the inflammation and reduced the thickness of the ear in a dose-dependent manner, indicating that IL-2/SD-01 can suppress the T cell immunity (Fig. 4b).

To demonstrate that the immuno-suppressive capability above is not restricted to a particular antigen, mice immunized with ovalbumin (OVA) were treated with IL-2/SD-01 (Fig. 4c). We analyzed the different immune cell subsets within the spleens by flow cytometry (Supplementary Fig. 2). Compared with PBS, IL-2/SD-01 induced more Treg cells for total cell counts and the percentage within CD4^+^ T cells (Fig. 4d,e) and reduced the abundance of follicular helper T (Tfh) cells and its percentage within CD4^+^ T cells (Fig. 4f, g). As a result, the total cell counts and the percentage of germinal center B (GCB) cells within B cells were reduced (Fig. 4h,i). To further demonstrate the advantage of IL-2/SD-01 over the established non-covalent complex of IL-2 and anti-IL-2 antibody, we incubated IL-2 with F5111.2 in the molar ratio of 2:1 and administered the mixture in mice in the same way of IL-2/SD-01. IL-2/F5111.2 could stimulate the proliferation of Treg cells, in agreement with previous report, but IL-2/SD-01 was more potent than IL-2/F5111.2 in Treg induction (Fig. 4d, e, j). Overall, IL-2/SD-01 not only boosts the proliferation of Treg cells but also is immuno-suppressive in immune-challenged mice.

### IL-2/SD-01 ameliorated disease onset and progression in rat Ulcer Colitis (UC) and mouse systemic lupus erythematosus (SLE) models

**Fig. 5:**
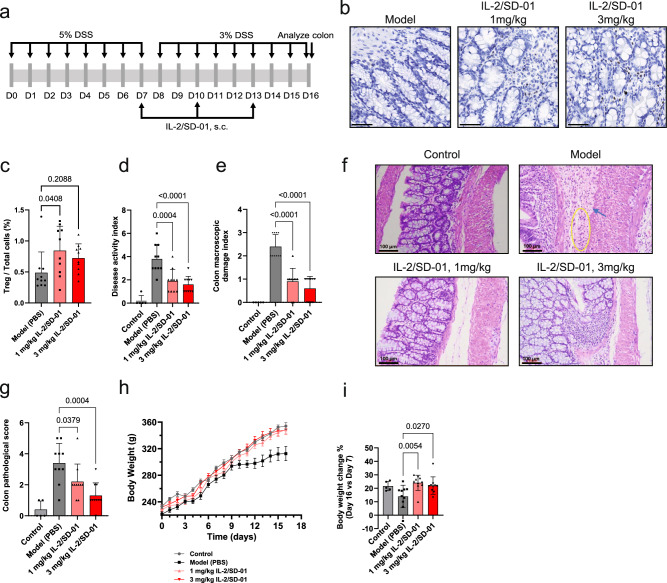
IL-2/SD-01 reduces the disease severity in a rat DSS-induced UC model. **a** Schematic of the experimental design of rat ulcerative colitis (UC) study. Rats were subjected to 5% (w/v) dextran sodium sulfate (DSS) in their drinking water for 8 days to induce colitis and then maintained by 3% DSS every day. Beginning on day 7, rats were treated Q3D for 10 days with PBS or IL-2/SD-01. Rats were sacrificed on day 16. **b** Immunohistochemistry staining of Foxp3 in colons of PBS- or IL-2/SD-01-treated rats. Foxp3^+^ Treg cells, black dots. Scale bars = 50 μm. **c** Quantification of the percentage of Foxp3^+^ Treg cells within total cells in immunohistochemistry-stained colons. **d** The disease activity index (DAI) in each treatment group at the end of the experiment is shown. **e** The colon macroscopic damage index (CMDI) in each treatment group at the end of the experiment is shown. **f** Representative images of colon tissues by hematoxylin and eosin (H&E) staining. The changes of colon were not found in control group, while necrosis in basal tissue (blue arrow) was found in model group, accompanied with severe ulceration and infiltration of inflammatory cells including neutrophils and monocytes (yellow circle). Mild inflammatory cell infiltration was found in both IL-2/SD-01-treated groups. Scale bars = 100 μm. **g** The quantification of colon pathological scores based on H&E staining of colon tissues. **h** Body weight was measured every day and means ± SEM are plotted. **i** The percentage changes of body weight from day 7 to day 16 in each treatment group were quantified. For all the above panels except being noted, means ± SD and individual values are plotted. Statistical significance of the differences between model group and the IL-2/SD-01-treated groups was determined by one-way ANOVA with Dunnett’s multiple comparisons. *n* = 10 animals in model and IL-2/SD-01-treated groups; *n* = 5 animals in control group. PBS, gray bar and closed square; 1 mg/kg IL-2/SD-01, light red bar and closed upward triangle; 3 mg/kg IL-2/SD-01, dark red bar and closed downward triangle.

To explore the therapeutic potential of IL-2/SD-01, we assessed its efficacy in a rat model of UC (Fig. 5a). Drinking water containing dextran sodium sulfate (DSS) can induce the inflammation of colon tissue in rats, which mimics the phenotype of UC in human patients. At the end of the experiment, compared with PBS-treated model group, rats treated with 1 mg/kg IL-2/SD-01 exhibited a substantial increase in the Treg infiltration in the colon tissues, consistent with the proliferation of peripheral Treg cells (Fig. 5b, c). As a result, we observed the reduction in disease severity, including dose-dependently ameliorated disease activity index (DAI, Fig. 5d), colon macroscopic damage index (CMDI, Fig. 5e) and colon pathology (Fig. 5f, g) in the IL-2/SD-01-treated animals. Meanwhile, IL-2/SD-01 maintained the weight gain, which was inhibited in PBS-treated model group (Fig. 5h, i). Our results suggest the therapeutic potential of IL-2/SD-01 in UC.

**Fig. 6:**
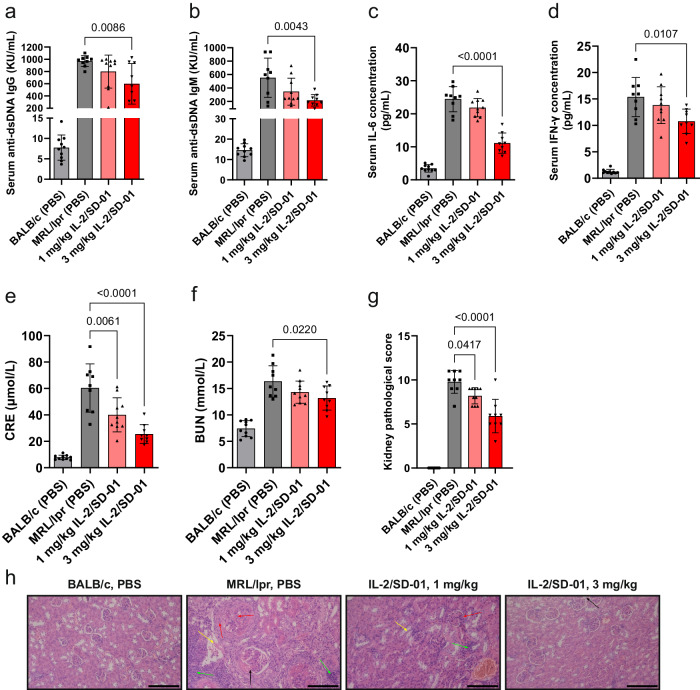
IL-2/SD-01 ameliorates the disease progression in MRL/*lpr* mice (spontaneous SLE). MRL/*lpr* mice at 8-week age (*n* = 10 animals) were treated with PBS, 1 mg/kg or 3 mg/kg IL-2/SD-01 subcutaneously every three days for 8 weeks. Mice were sacrificed at the end of the experiment. Anti-dsDNA IgG (**a**) and IgM (**b**), IL-6 (**c**), IFN-γ (**d**), serum creatinine (CRE) (**e**), blood urea nitrogen (BUN) (**f**) within serum were measured. **g** Kidney pathological score was assessed based on immunohistological images of kidney tissues. For all the above panels, means ± SD and individual values are plotted. Statistical significance of the differences between MRL/lpr model group and the IL-2/SD-01-treated groups was determined by one-way ANOVA with Dunnett’s multiple comparisons. *n* = 10 animals. PBS, gray bar and closed square; 1 mg/kg IL-2/SD-01, light red and closed upward triangle; 3 mg/kg IL-2/SD-01, dark red and closed downward triangle. **h** Representative images of kidney tissues by H&E staining. The changes of kidney were not found in control group (BALB/c, PBS), while glomeruli depression (black arrow), intumescentia and hyperplasia (red arrows), renal tubular injury (yellow arrow) accompanied with inflammatory cell infiltration (green arrow) were found in model group, which were alleviated in IL-2/SD-01-treated groups. Scale bar = 100 μm.

We also tested the efficacy of IL-2/SD-01 in a mouse model of SLE. MRL/*lpr* mice spontaneously develop an autoimmune disease resembling SLE due to a mutation in *Fas* gene that promotes survival of self-reactive lymphocytes. The phenotype includes accumulation of autoantibodies, elevated inflammation, renal dysfunction and so on. When treated with IL-2/SD-01, the serum anti-dsDNA IgG and IgM level dropped (Fig. 6a, b), consistent with previous finding that IL-2/SD-01 suppresses humoral immunity. Likewise, the levels of cytokines that induce inflammation, including IL-6 and IFN-γ (Fig. 6c, d), were reduced. IL-2/SD-01 also promoted the clearance of serum creatinine (CRE) and blood urea nitrogen (BUN) (Fig. 6e,f), and improved the kidney pathological phenotype (Fig. 6g,h), suggesting the recovery of certain renal functions. Collectively, IL-2/SD-01 suppresses the immunity and demonstrates great therapeutic potential in a broad spectrum of autoimmune diseases.

### IL-2/SD-01 extended the half-life of IL-2 and selectively activated Treg cells in non-human primates with favorable safety profile

**Fig. 7:**
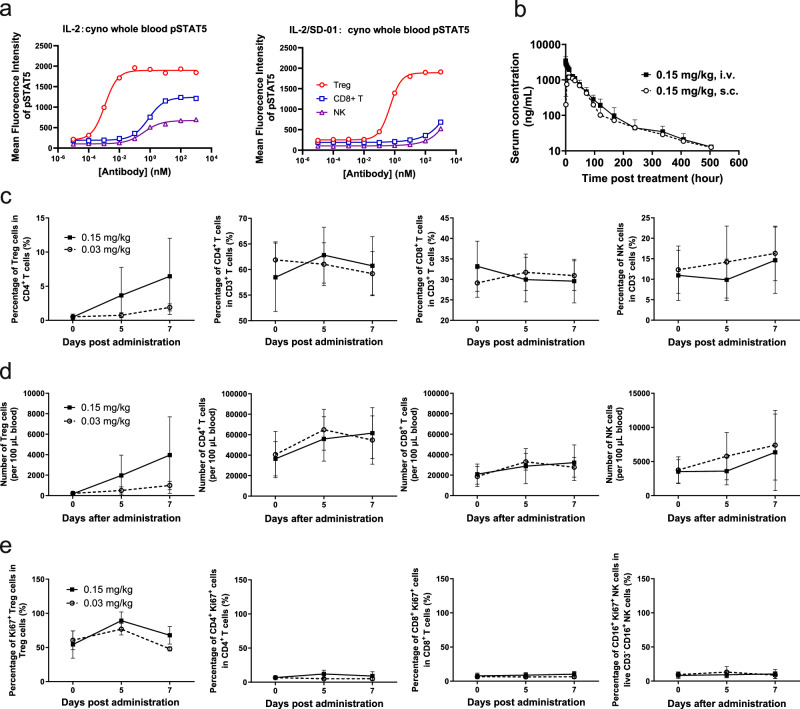
IL-2/SD-01 extends IL-2’s half-life and selectively activates Treg cells in nonhuman primates. **a** STAT5 phosphorylation responses of the indicated cynomolgus cell subsets to serial dilutions of IL-2 or IL-2/SD-01. Treg cells, red open circle; CD8 + T cells, blue open square; NK cells, purple open triangle. **b** Pharmacokinetics of IL-2/SD-01 in cynomolgus monkeys. A single dose of 0.15 mg/kg IL-2/SD-01 was administered intravenously (i.v.) (*n* = 6 animals, 3 per sex, closed square) or subcutaneously (s.c.) (n = 6 animals, 3 per sex, open circle). Serum was collected at the indicated time points and the level of IL-2/SD-01 was determined by ELISA. Data represent mean ± SD. **c**–**e** Cynomolgus monkeys were administered with subcutaneous injection of 0.03 (open circle) or 0.15 mg/kg (closed square) IL-2/SD-01. Blood were collected at different time points after the first injection. Cells were stained with antibodies against CD3, CD4, CD8, CD16, CD25, FoxP3 and Ki67, and gated on Treg, CD4^+^ T, CD8^+^ T and NK cells. The percentage of Treg cells within CD4^+^ T cells, the percentage of CD4^+^ T cells within CD3^+^ T cells, the percentage of CD8^+^ T cells within CD3^+^ T cells and the percentage of NK cells within CD3^−^ T cells are shown (**c**); the total cell counts of Treg, CD4^+^ T, CD8^+^ T and NK cells are shown (**d**); the percentages of Ki67^+^ cells within Treg, CD4^+^ T, CD8^+^ T and NK cells are also shown (**e**). Data are mean ± SD. *n* = 6 animals.

Human IL-2 binds mouse IL-2Rs with lower affinities than their human counterpart receptors. The effect of human IL-2 in mice might be underestimated. To better demonstrate the therapeutic relevance of IL-2/SD-01, we first performed ex vivo pSTAT5 induction assay in cynomolgus whole blood. Treg, CD8^+^ T and NK cells were divided on the basis of the expression of CD3, CD4, CD8, CD16, CD25 and Foxp3 (Supplementary Fig. 3). IL-2/SD-01 stimulated cynomolgus Treg but not CD8^+^ T and NK cells at intermediate concentration, in a similar fashion to the situation in human PBMC (Fig. 7a), consistent with a high degree of homology between human and cynomolgus IL-2Rs. The result suggests that cynomolgus monkey is a relevant species for preclinical pharmacokinetic, pharmacodynamic and safety studies to facilitate the initiation of a first-in-human clinical trial of IL-2/SD-01.

We administered cynomolgus monkeys with a single dose of 0.15 mg/kg IL-2/SD-01 intravenously (i.v.) or subcutaneously (s.c.). Compared with the pharmacokinetics in mice, IL-2/SD-01 showed an even longer half-life of ~61.1 h (i.v.) or ~72.7 h (s.c.) and a relative bioavailability of 67.1% after being subcutaneously administered (Fig. 7b). Thus, IL-2/SD-01 can prolong the serum half-life of IL-2 in cynomolgus monkeys.

To further assess the long-term safety profile of IL-2/SD-01, we conducted a multiple-dose toxicity study in three groups (*n* = 10 per group, 5 males and 5 females) of cynomolgus monkeys, which received 5% glucose solution (vehicle), 0.03 or 0.15 mg/kg IL-2/SD-01 subcutaneously once a week for 13 weeks, followed by a 6-week recovery period without any treatment. We quantified the abundance and proliferation status of different blood cell subsets pre- and post-treatment of the first injection by flow cytometry analysis. The proportion of Treg cells in CD4^+^ T cells and their cell counts increased on day 5 and 7 after IL-2/SD-01 dosing in IL-2/SD-01-treating groups (Fig. 7c, d). The proportions of Treg cells in 0.03 and 0.15 mg/kg groups on day 7 were increased by 3.6 and 13.4 folds, respectively, compared to those of pre-dose, whereas the proportions of CD4^+^ T, CD8^+^ T, and NK cells remained almost constant (Fig. 7c). The cell counts of Treg cells in 0.03 and 0.15 mg/kg groups on day 7 were increased by 4.4 and 22.3 folds, respectively, whereas the change in the cell counts of CD4^+^ T, CD8^+^ T, and NK cells was no more than 2 folds (Fig. 7d). The expression of Ki67 increased in Treg cells on day 5 and dropped on day 7, but kept unchanged in CD4^+^ T, CD8^+^ T, and NK cells during the first cycle, suggesting that Treg cells were proliferating, consistent with the change in the abundance of different cell subsets (Fig. 7e). For each group, 6 animals (3 males and 3 females) were dissected at the end of the 13-week treatment and 4 animals (2 males and 2 females) were dissected at the end of the recovery period. During the entire study, no mortality and major drug-related adverse effects were observed in any group, except that minimal myometrial/endometrial multifocal perivascular mononuclear cell infiltration was observed microscopically in one female of each IL-2/SD-01-treating group at the end of the 13-week treatment (Supplementary Fig. 4a, b). The infiltration did no harm to animals’ health and was not detected at the end of the recovery period, which was therefore not considered to be an adverse effect. We conclude that 0.03 mg/kg could be a minimal effective dose, while 0.15 mg/kg could be the no observed adverse effect level in this study. Overall, IL-2/SD-01 is a long-lasting IL-2 modified by the covalently-linked antibody that retains the biased activity towards Treg cells and demonstrates favorable long-term safety profile in non-human primates.

## Discussion

Much interest has grown in the treatment of autoimmune diseases with low-dose IL-2. Nevertheless, it is widely agreed that the dual functions of IL-2 to stimulate immune-suppressive Treg cells and immune-activating Teff and NK cells, along with its short half-life, hamper the clinical applications of low-dose IL-2. Therefore, many efforts have been made to reinforce the bias of wild-type IL-2 to Treg cells over Teff and NK cells.

One strategy is to introduce mutations on IL-2 that reduce affinity towards IL-2Rβ or IL-2Rγ and thus to increase relative dependence on IL-2Rα and Treg selectivity^22^. However, cytokine muteins may alter the strength of receptor dimerization and exhibit divergent biological behaviors from wild-type cytokines^23–25^. This creates a clinical uncertainty, especially given that some functions of wild-type cytokines have been proved beneficial. Moreover, cytokine muteins may raise concerns about immunogenicity, a more highlighted issue for chronic therapy of autoimmune diseases.

To overcome these limitations, an alternative strategy was developed in which wild-type IL-2 forms non-covalent complex with anti-IL-2 antibodies. The antibodies sterically block IL-2 binding to IL-2Rβγ and therefore disfavor the activation of Teff and NK cells by IL-2; the antibodies also sterically or allosterically interfere with IL-2 binding to IL-2Rα and release IL-2 to IL-2Rβγ upon contact with IL-2Rα on Treg cells^17,18^. In this study, we discovered an antibody SD-01 bearing a similar property that completely blocked IL-2 binding to IL-2Rβγ but slightly reduced IL-2 binding to IL-2Rα. As expected, SD-01 reduced the activity of IL-2 more on CD8^+^ T and NK cells than on Treg cells. However, the non-covalent nature of IL-2/antibody complex dictates the inherent instability of the complex and persistent dissociation between IL-2 and the anti-IL-2 antibody. It is challenging to identify the dosing regimen and optimal ratio between IL-2 and anti-IL-2 antibody for such a therapeutic with two interacting components whose concentrations keep changing in human body. A more complicating scenario stems from the divergent pharmacokinetics between IL-2 and the anti-IL-2 antibody. As IL-2 is consumed by Treg cells and anti-IL-2 antibody dissociates, the long half-life of anti-IL-2 antibody may cause its own accumulation. It has been reported that the administration of free Treg-biasing anti-IL-2 antibodies, such as JES6-1 and UFKA-20, resulted in decreased percentage of Treg cells^9,18^. Thus, an excess of anti-IL-2 antibody may generate opposing effects.

Previous study has demonstrated in principle that mouse IL-2 can be covalently fused to anti-mouse IL-2 antibody to stabilize their association^19^. But the generation of such covalent complex required extensive engineering efforts based on structural information and it was uncertain whether the strategy can be applied to human IL-2. In the current study, we generated a single-agent covalently-linked human IL-2 and anti-human IL-2 antibody complex that selectively activates Treg cells. In fact, the affinity between IL-2 and SD-01 is one order of magnitude lower than that between IL-2 and JES6-1, F5111.2 or UFKA-20, making SD-01 more suitable for such molecular design in which intramolecular interaction between IL-2 and anti-IL-2 antibody should not be too strong for IL-2 to be released upon contact with Treg cells. Otherwise, additional destabilizing mutations on the interface between IL-2 and anti-IL-2 antibody would be necessary^19^. We noticed a recent report of a similar Treg-biasing covalently-linked IL-2/antibody complex, which contained both monomeric and higher-order oligomeric species^26^. The authors admitted that the heterogeneity of the molecule made it difficult to advance the clinical development and that further optimization of sequence was warranted. We screened a variety of different antibodies for this type of design and did not observe the developability issues in our final molecule, highlighting the importance of the correctly-chosen anti-IL-2 antibody in drug developability. We also found that the relative fusion position of IL-2 to anti-IL-2 antibodies (i.e., IL-2 tethering to N terminus of either heavy or light chain of the antibody) and the linker length between them control the purity and activity of the covalent complex, potentially reflecting the particular binding geometry between IL-2 and anti-IL-2 antibody. These varying factors, including antibody epitope, affinity to IL-2, fusion position and linker length, should be compatible with each other to spatially allow for intramolecular engagement and a matrix screening approach might be necessary, especially when the complex structure is not available.

Although SD-01 partially dampens the monovalent interaction between IL-2 and IL-2Rα (Fig. 2d, e), either sterically or allosterically, the bivalency of IL-2 within IL-2/SD-01 compensates the reduced affinity and IL-2/SD-01 exhibits enhanced apparent avidity towards IL-2Rα, compared to that between wild type IL-2 monomer and IL-2Rα. On the other hand, the complete blockage of the interaction between IL-2 and IL-2Rβγ by SD-01 cannot be counteracted by IL-2 bivalency and IL-2/SD-01 cannot bind IL-2Rβγ in the absence of IL-2Rα. Presumably, IL-2/SD-01 binds the constitutively-expressed IL-2Rα on Treg cells, which in turn weakens the interaction between SD-01 and IL-2 and allows for the engagement of IL-2Rβγ with IL-2, as exemplified by JES6-1, F5111.2 and UFKA-20^10,17,18^. The bivalent nature of our design increases its recruitment to IL-2Rα on Treg cells and enables the triggering ability of IL-2Rα.

It should be noted that the fold difference between the concentrations required to activate Treg and CD8^+^ T or NK cells is quite similar for wild-type IL-2 and IL-2/SD-01, at least in human PBMC pSTAT5 activation assay. A previous study reported that a fusion protein of mouse IL-2 and mouse IL-2Rα selectively stimulated Treg cells, in which IL-2 interacted with IL-2Rα in trans to form inactive head-to-tail dimers that slowly dissociate into active monomers^27^. It is quite possible that IL-2/SD-01 similarly transits between the intramolecular IL-2/SD-01 associated and dissociated states, with the dissociated state responsible for the activation of Treg, Teff and NK cells. In the associated state, IL-2 is sequestered by SD-01 and IL-2/SD-01 can still bind IL-2Rα, but it seems that the cooperativity between the binding of IL-2/SD-01 to IL-2Rα and IL-2Rβγ doesn’t exist. In the dissociated state, IL-2 is fully released from SD-01 to deliver 100% pSTAT5 activity at plateau. The equilibrium between the associated and dissociated states determines the potency of IL-2/SD-01 to Treg, Teff and NK cells, while the bias towards Treg cells is retained. The utilization and consumption of IL-2/SD-01 in the dissociated state by immune cells would further shift the equilibrium towards the dissociated state, while the inactive associated state constitutes the reservoir of the active dissociated state. Further biophysical characterization, beyond the scope of the current study, is required to test the model.

Short half-life demands frequent dosing of low-dose IL-2. In contrast, a single dose of IL-2/SD-01 elicited strong proliferation of Treg cells without provoking CD8^+^ T and NK cells in mice and non-human primates. A prolonged serum persistence due to increased molecular size, neonatal Fc receptor-mediated recycling, less Teff and NK-cell-mediated drug disposition and subcutaneous administration could be important contributing factors. Despite the stronger potency of IL-2 in pSTAT5 assay, IL-2/SD-01 is better able to maintain serum concentration high enough to ensure efficient Treg activation. For IL-2 to achieve similar level of activation, a higher and more frequent dosing may be required, at the risk of toxicity. In fact, daily administration of low-dose IL-2 in patients sometimes resulted in fever, chills, influenza-like symptoms, headaches, dizziness, and arthralgia/myalgia, indicating an unmet medical need for IL-2-based therapy^28^. Our multiple-dose toxicity study in cynomolgus monkeys showed that IL-2/SD-01 can be administered weekly for 13 weeks without causing obvious adverse effect, suggesting that a potent IL-2-based therapy with less-frequent dosing and long-term safety profile is possible.

The limitation of this study is that the efficacy of IL-2/SD-01 was entirely based on Treg activation and was only tested in limited animal models, such as UC and SLE. The genetic background and the disease causes of human patients could be very diverse. It has been reported that IL-2 mutein can ameliorate pathological inflammation in a DSS-induced but not T-cell transfer colitis model, because IL-2Rα^+^CD8^+^ T cells exist in large amount in inflammatory lesions in the latter model^23^. Similar to IL-2 muteins, IL-2/SD-01 is also not immune from the activation of IL-2Rα^+^CD8^+^ T cells. The caveat of that study is that persistent expression of IL-2Rα on Teff and NK cells may not reflect the typical situation during disease progression. Instead, the expression of IL-2Rα on Teff and NK cells is more likely to be transient. But these findings highlight the importance of the timing of the treatment and the immunological conditions of the patients. Treatment of IL-2/SD-01 should be avoided for the patients in acutely inflammatory conditions with active T cell response. Further translational studies should be conducted to select the right patient population and the administration scheme.

In the rat UC model, we observed a large increase of Treg cells in the colons of the animals treated with lower dose of IL-2/SD-01, a result reminiscent of a previous study that short-term treatment of IL-2/anti-IL-2 antibody complex induced the long-term homing of Treg cells and the formation of inducible tertiary lymphoid structures (iTLS) within the lung allografts and facilitated the lung allograft acceptance in mice^29^. We did not evaluate the time course of Treg residence in the colons, but the gastrointestinal tract might affect the residence duration of Treg cells differently from the lung allograft, considering that the immune cells in the gastrointestinal tract are exposed to and reshaped by commensal microbiota and/or food all the time. Indeed, it has been reported that Treg cells are continuously generated and replaced in small intestine^30^. Treg cells are heterogenous and different Treg subsets in gut or skin tissues could have diverse turnover rates in lymph nodes, ranging from days to weeks and months^31^. Further studies are required to assess the long-term homing of Treg cells in colons and their role in the control of UC pathology.

Collectively, we engineered a single-agent fusion of human IL-2 and a fully human anti-IL-2 antibody, termed IL-2/SD-01, that preferentially stimulates Treg cells in mice and non-human primates in vivo as well as in human ex vivo. We demonstrated its suppression on the immunity, its effectiveness in rodent models of UC and SLE and its favorable long-term safety profile. Investigational New Drug (IND) application of IL-2/SD-01 has been approved and a first-in-human clinical trial in SLE and other autoimmune diseases is ongoing.

## Methods

### Protein expression and purification

Human IL-2, human IL-2Rα, human IL-2Rβγ and their biotinylated counterparts were all purchased commercially. For the anti-IL-2 antibody SD-01, the heavy and light chains were generated by fusing the variable regions with the constant regions of human IgG1 and kappa chains and were separately cloned into the pcDNA3.4 plasmids with signal peptides at the very N terminus to allow extracellular secretion. For IL-2/SD-01 fusion protein, the human IL-2 was further fused to the N terminus of the light chain of SD-01 through a (GGGGS)*5 linker, while the heavy chain remained the same. The heavy and light chains were mixed with a ratio of 2:3 and electro-transfected into CHO cells. The expression was allowed for 4-6 days and the supernatant was collected and purified to >95% homogeneity with a protein-A affinity column and buffer-exchanged to PBS. Purity was verified by SDS-PAGE and size exclusion chromatography.

### Enzyme-linked immunosorbent assay

96-well high-binding flat-bottom microtiter plates were coated overnight at 4 °C with 0.5 μg/mL Fc-tagged human IL-2Rα (Novoprotein, CJ78) or 8 μg/mL Fc-tagged human IL-2Rβ (Novoprotein, CJ82) in PBS. Plates were washed with PBST (PBS containing 0.05% Tween-20) and blocked with 1% (w/v) bovine serum albumin at room temperature for 1 h. Serial dilutions of anti-IL-2 antibody were mixed with laboratory-prepared biotinylated Fc-tagged IL-2 (final concentration 1 μg/mL and 5 μg/mL for IL-2Rα and IL-2Rβ, respectively) for 15 min. 30 μL of the mixture was added to the plates at room temperature for 1 h. The plates were washed with PBST and incubated with HRP-conjugated neutravidin (1:6000) at room temperature for 1 h. Plates were washed with PBST and developed with TMB substrate. The reaction was terminated using 2 M HCl and read immediately at 450 nm.

### Surface plasmon resonance

Human Antibody Capture Kit (Cytiva, BR100839) and CM5 chip (GE Healthcare) were used to make anti-human IgG (Fc) chip for the affinity detection of human IL-2 (Sino Biological, GMP-11848-HNAE) with SD-01. SD-01 in HBS-EP^+^ buffer (Cytiva, BR100669, 33372) was injected over the chip surface for 30 s (speed: 10 μL/min) as ligand. Then the 2-fold dilution gradient (3.9–250 nM) of human IL-2 was injected as analyte with the speed of 30 μL/min. The chip surface was regenerated after each injection by washing with Glycine 1.5 (Cytiva, BR100354) for 30 seconds (speed: 30 μL/min).

His Capture Kit (GE Healthcare, 29234602) and CM5 chip (GE Healthcare) were used to make anti-His tag chip. The his-tagged human IL-2Rα (Sino Biological, 10165-H08H) or IL-2Rβγ (ACROBiosystems, ILG-H5283) was injected over the chip surface and captured as ligand. The 2-fold dilution gradient of tag-free IL-2 (1.56–100 nM, R&D, 202-IL-050/CF), Fc-tagged IL-2 (0.7825–25 nM, ACROBiosystems, IL2-H5269) or IL-2/SD-01 (0.0976–3.125 nM) was injected as analyte with the speed of 30 μL/min. The surface was regenerated after each injection by washing with regeneration buffer (Cytiva, BR100354) for 30 seconds (speed: 30 μL/min) and binding affinities were measured by surface plasmon resonance on a Biacore 8 K (GE Healthcare).

### In vitro pSTAT5 activation in human PBMCs and whole blood of cynomolgus monkey

The 10-fold dilution gradient (0.00001 ~ 1000 nM) of human IL-2 and IL-2/SD-01 or the indicated concentrations of non-covalent complex of IL-2 and anti-IL-2 antibodies were mixed with human PBMC (1 × 10^6^ cells per well) and incubated for 15 min at 37 °C, 5% CO_2_. 100 µL Cytofix Fixation Buffer (BD, lot:554655) was added for 15 min at 37 °C, 5% CO_2_, followed by 4 °C fixation for 15 min. Cells were collected by centrifugation at 500 g, washed twice with FACS buffer (2% FBS in PBS), then resuspended by 150 µL/well Phosflow Perm Buffer III (BD, lot: 558050), and membranes were permeabilized at −20 °C overnight. On the second day, cells were collected and incubated at 4 °C for 15 min with Human BD Fc Block (BD, lot: 564219) after being washed twice with FACS buffer. The cells were collected, resuspended with staining antibodies and then incubated at 4 °C for 60 min. Cells were washed twice with FACS buffer, then resuspended with 150 µL FACS buffer for FACScelesta (BD) detection. The induction of pSTAT5 was assessed in CD3^+^CD4^+^CD25^high^ Foxp3^+^ Treg cells, CD3^+^CD8^+^ T cells and CD3^−^CD56^+^ NK cells by FlowJo^TM^ (BD). Antibodies and reagents are listed in Supplementary Table 1.

The 10-fold dilution gradient (0.00001 ~ 1000 nM) of human IL-2 or IL-2/SD-01 were mixed with whole blood of cynomolgus monkey, and 1× BD PhosFlow Lyse/Fix buffer was added for 10 min in 37 °C after an incubation of 15 min in 37 °C, 5% CO_2_, and the following procedures were in accordance with human PBMCs section. The induction of pSTAT5 was assessed in CD3^+^CD4^+^CD25^high^ Foxp3^+^ Treg cells, CD3^+^CD8^+^ T cells and CD3^−^CD16^+^ NK cells. Antibodies and reagents are listed in Supplementary Table 2.

### Pharmacokinetics and pharmacodynamics in mice

For pharmacokinetics study, wild type female BALB/c mice at the age of 6–8 weeks were administered with a single dose of 0.7 mg/kg (5 mL/kg) IL-2/SD-01 subcutaneously (*n* = 12). The animals were randomly divided into four groups, whose blood was collected sequentially at pre-dose, 1 h, 2 h, 4 h, 8 h, 12 h, 24 h, 48 h, 72 h, 96 h, 120 h and 144 h, with the blood of only one group collected per time point. Blood samples were incubated at room temperature for 30 min and centrifuged to obtain serum sample (2000 g, 5 min under 4 °C). High-binding microplates (Corning, Cat. 9018) were coated with 50 µL/well 2.5 µg/mL goat anti-human IgG Fc antibody (Rockland, Cat. 609-101-017) at 4 °C overnight. The plates were washed three times with PBST and blocked for 1 h at room temperature. 50 µL IL-2/SD-01 standard samples and blood samples (diluted with PBS, 1% BSA, 0.05% Tween-20, 0.05% proclin 300) were added after washing and incubated for 1 h at 37 °C. Wells were washed three times with PBST and 100 µL HRP-conjugated anti-human IgG (Sigma, Cat. A0293, 1:5000 dilution) was added and incubated for 1 h at 37 °C. The plates were washed three times with PBST and were developed with 100 µL TMB substrate (InnoReagents, Cat. TMB-S-003) at room temperature for 6 min and stopped by adding 2 M H_2_SO_4_. Absorbance at 450–630 nm was read using a microplate reader (SpectraMax plus384).

For pharmacodynamics study, wild type female BALB/c mice at age of 6–8 weeks were administered with a single dose of 0.7 mg/kg IL-2/SD-01 subcutaneously (*n* = 3). At pre-dose, day 2, 3, 4, 5 and 7 days after treatment, whole blood cells were collected. PBMCs were resuspended with 100 μL/well staining mixture containing antibodies against CD3, CD4, CD8 and CD25. The samples were incubated at 4 °C for 30 min before being washed twice with PBS containing 1% FBS. The cells were then fixed and permeabilized with True-Nuclear^TM^ Transcription Factor Buffer Set for 1 h and intracellularly stained with antibodies against Foxp3 and Ki67 for 1 h at room temperature. The samples were washed twice with PBS and resuspended with 500 μL PBS before being analyzed on FACSCelesta (BD). Treg cells were identified as CD3^+^CD4^+^CD25^+^Foxp3^+^ cells, while T_conv_ cells and CD8^+^ T cells were gated on CD3^+^CD4^+^CD25^-^ and CD3^+^CD4^−^CD8^+^ cells, respectively. Antibodies and reagents are listed in Supplementary Table 3.

### Mouse model of delayed-type hypersensitivity

Male ICR mice at age of 6 weeks (five per cohort) were sensitized on day 0 through topical application of 50 μL 1% (w/v) DNFB (Sigma, 42085-50 G) solution to the shaved abdomen. Challenge was performed on day 5 through application of 10 μL 0.5% (w/v) DNFB to the inner and outer surfaces of the right ear (total 20 μL). On day 6, thickness of both left and right ears were measured. Both left and right ear tissues were also collected with 8 mm-diameter hole puncher and the weight was measured. The covalently linked IL-2/IL-2 antibody complex was administered subcutaneously on day -2 and 3.

### Inhibition of humoral immunity in mice immunized with ovalbumin (OVA)

OVA (Sigma, A5503) was dissolved in PBS to the concentration of 0.5 mg/mL. 8 mL of OVA and 8 mL of complete freund’s adjuvant (CFA) (Sigma, F5881) were mixed into emulsion. Male C57BL/6 mice at the age of 6–8 weeks (six per cohort) were immunized on day 0 with 200 μL of OVA/CFA emulsion intraperitonealy. 0.7 mg/kg IL-2/SD-01 or equal amount of PBS was administered subcutaneously on day 3 and 8. For the group of IL-2 + F5111.2, 0.57 mg/kg F5111.2 and 0.12 mg/kg IL-2 (corresponding to a molar ratio of 1:2) were pre-incubated for 30 min and administered subcutaneously on day 3 and 8. On day 10, spleens were collected and Treg and Tfh (follicular T helper cells) and germinal center B cells were analyzed by fluorescence-activated cell sorting (FACS). Treg cells were identified as CD4^+^CD25^+^Foxp3^+^CXCR5^low^, while Tfh and germinal center B cells were gated on CD4^+^PD-1^high^Foxp3^-^CXCR5^high^ and B220^+^GL7^+^Fas^+^, respectively. Antibodies and reagents are listed in Supplementary Table 4.

### Rat model of dextran sulfate sodium salt (DSS)-induced UC

Specific-pathogen-free (SPF) male Sprague-Dawley rats at the age of 6–8 weeks were provided by the Beijing Vital River Laboratory Animal Technology Co., Ltd. Ulcerative colitis was induced by oral administration of 5% (w/v) DSS (Yeasen Biotechnology, D1127960) dissolved in drinking water for eight consecutive days, and then maintained by 3% DSS to avoid self-cure. Except for the DSS-untreated control group (*n* = 5), UC rats were randomly divided into three groups of 10 individuals each: PBS-treated model group, 1 mg/kg IL-2/SD-01 group and 3 mg/kg IL-2/SD-01 group. More specifically, rats in model group were given only PBS by subcutaneous injection twice a week for ten days. IL-2/SD-01 group were given IL-2/SD-01 in PBS buffer subcutaneously twice a week for ten days based on body weight.

Disease activity index (DAI) was determined by combining scores of body weight loss, stool looseness and stool bleeding. Each score was determined as follows: change in body weight loss (0: 0%; 1: 1–5%; 2: 5–10%; 3: 10–15%; 4: >15%), stool looseness (0: normal; 1: loose stool, small amount; 2: loose stool, large amount; 3: diarrhea, small amount; 4: diarrhea, large amount), stool bleeding (0: negative; 1: fecal occult blood, weak positive; 2: fecal occult blood, positive; 3: hematochezia, small amount; 4: hematochezia, large amount).

Colon macroscopic damage index (CMDI) was evaluated as follows: 0: no inflammation and no ulceration; 1: no ulcer, mild mucosal congestion and edema; 2: congestion, edema and ulcers; 3: severe congestion and edema, inflammation, ulcer longitudinal length of less than 1 cm; 4: severe congestion and edema, inflammation, ulcer longitudinal length of more than 1 cm.

The colon tissue was hematoxylin and eosin (H&E) stained to evaluate the pathological score as follows: 0: normal; 1: mild inflammatory cell infiltration in mucosa, edema, mucosal erosion but intact muscularis mucosae; 2: the characteristics of 1 observed in more than 50% of the tissue; 3: moderate inflammation and edema, mucosal erosion and inflammatory cell infiltration in muscularis mucosae; 4: the characteristics of 3 observed in more than 50% of the tissue; 5: ulcers accompanied with large amount of neutrophil and monocyte infiltration, necrosis in muscularis mucosae; 6: the characteristics of 5 observed in more than 50% of the tissue.

The body weight was measured every day for each rat and the percentage change in body weight between day 7 and day 16 was calculated based on the equation: percentage change = (Body weight_D16_ – Body weight_D7_) / Body weight_D7_ * 100%.

For immunohistochemistry staining of the colon tissues, the tissue was first fixed with formalin. The antigen unmasking of the formalin-fixed paraffin-embedded (FFPE) tissue was done by incubating samples in BOND™ Epitope Retrieval Solution 2 (Leica Biosystems, Cat. # AR9640) for 20 min at 100 °C. Endogenous peroxidase activity was quenched by incubation of the samples in pre-primary peroxidase inhibitor (BOND™ Polymer Refine Detection; Leica Biosystems, Cat. # DS9800) for 5 min at ambient temperature (20–25 °C). Sample slides were incubated with the FoxP3 primary antibody (Abcam, Cat. # ab215206), diluted to 2.0 μg/mL (1:200) in BOND Primary Antibody Diluent (Leica Biosystems, Cat. # AR9352) for 30 min at ambient temperature. Incubation of the sample slides with the secondary polymer anti-rabbit poly–horseradish peroxidase-IgG was done for 8 min, followed by incubation with the 3,3′-diaminobenzidine tetrahydrochloride hydrate (DAB) chromogen (BOND™ Polymer Refine Detection; Leica Biosystems, Cat. # DS9800) for 10 min at ambient temperature. After washing off the excess DAB, sample nuclei were counterstained with hematoxylin (BOND™ Polymer Refine Detection; Leica Biosystems, Cat. # DS9800) for 5 min at ambient temperature. Images were scanned with 3DHISTECH scanner. Quantification of FoxP3^+^ cells was performed with HALO software (HALO, US). Not all colons contained the lymph nodes and therefore the lymph nodes in the colons and the Treg cells within them were excluded from the quantification.

### Mouse model of SLE

The mice were maintained under specific pathogen-free facility following institutional guidelines. The control group contained 10 female BALB/c mice, and female MRL/*lpr* mice were randomized into three groups of 10 individuals each: model group, 1 mg/kg IL-2/SD-01 group and 3 mg/kg IL-2/SD-01 group. Beginning at age of 8 weeks, mice in model group were given only PBS by subcutaneous injection every 3 days for 8 weeks. IL-2/SD-01 group were given IL-2/SD-01 in PBS buffer subcutaneously every 3 days for 8 weeks. Serum creatinine (CRE), blood urea nitrogen (BUN) and total urine protein were analyzed using assay kits from Nanjing Jiancheng Bioengineering Institute (lot: C011-2-1, lot: C013-2-1, lot: C035-2-1). Autoantibodies in serum (Anti-dsDNA IgG and IgM) and cytokines (IL-6 and IFN-γ) were detected using the mouse ELISA Kits (Alpha diagnostic, lot: 5120, lot: 5130; Dakewe Biotech, lot: 1210602, lot: 1211002, lot: 1210002), and all procedures were according to the manufacturer’s instructions. Kidney samples were fixed with 4% formaldehyde, dehydrated, immersed in wax, embedded in paraffin and finally cut into sections for H&E staining to evaluate the change of histopathology. The grading scheme was modified according to the method reported previously^32^. Basically, kidney lesions include glomerulonephritis, interstitial nephritis and vascular lesions. Glomerulonephritis was graded as follows: 1 + , mild proliferative; 2 + , multifocal proliferative with increased inflammatory cells; 3 + , diffusive proliferative; 4 + , proteinuria. Interstitial nephritis was graded as follows: 1 + , 1–3 small cluster (5–10 cells) of mononuclear cell infiltration; 2 + , mild mononuclear cell infiltration around tubules and small tubular atrophy; 3 + , moderate mononuclear cell infiltration and large tubular atrophy; 4 + , extensive mononuclear cell infiltration and tubular necrosis. Vascular lesions were graded as follows: 1 + , mononuclear cell infiltration around renal pelvis; 2 + , mononuclear cell infiltration around main arteries; 3 + , mononuclear cell infiltration around small arterial branches; 4 + , mononuclear cell infiltration extending into surrounding parenchyma and most vessels.

### Pharmacokinetics in cynomolgus monkeys

Cynomolgus monkeys at the age of 2–4.5 years were randomized into two groups of six animals each (3/sex) and administered with 0.15 mg/kg IL-2/SD-01, i.v. or 0.15 mg/kg IL-2/SD-01, s.c., respectively. Blood of the i.v. group were collected at pre-dose and 0, 1, 4, 8, 12, 24, 32, 48, 72, 96, 120, 168, 240, 336, 408, 504 h after administration, while the s.c. group were collected at pre-dose and 0.0833, 0.25, 1, 4, 8, 12, 24, 32, 48, 72, 96, 120, 168, 240, 336, 408, 504 h after administration. The serum concentrations of IL-2/SD-01 were detected by ELISA. Flat-bottom 96-well plates (Thermo Fisher Scientific) were coated overnight at 4 °C with rabbit anti-IL-2/SD-01 IgG. After the plates were washed with PBS containing 0.05% Tween 20 (Sigma-Aldrich), wells were blocked by 3% Milk-PBST for 1.5 h at 25 °C. 100 µL IL-2/SD-01 standard sample and blood sample (100× diluted by 3% Milk-PBST) were added after washing and incubated for 1 h at 25 °C. Wells were washed and 100 µL biotinylated goat anti-human IgG-heavy and light chain monkey absorbed antibody (Bethyl, lot: A80-319B-14/A80-319B) was added and incubated for 1 h at 25 °C. After an additional wash, plates were incubated with HRP-conjugated streptavidin (Thermofisher) for 1 h at 25 °C in the darkness. After the last wash, 96-well plates were developed with TMB Peroxidase EIA substrate (Solarbio) for 6 to 8 min and stopped by adding 1.8 M H_2_SO_4_ (Sinopharm chemical reagent Co., Ltd.). Absorbance at 450–630 nm was read using a microplate reader (MD sectramax plus384). WinNonlin Phoenix (8.1 version) was used for calculation of pharmacokinetic parameters.

### Long-term safety and pharmacodynamics study of IL-2/SD-01 in cynomolgus monkeys

30 cynomolgus macaques at the age of 3–4 years were randomized into 3 groups of 10 animals each (5 male and 5 female per group): vehicle group, 5% glucose solution; low-dose group, 0.03 mg/kg IL-2/SD-01; high-dose group, 0.15 mg/kg IL-2/SD-01. Individuals were subcutaneously treated with 5% glucose solution or IL-2/SD-01 once a week for 13 weeks followed by a recovery period of 6 weeks. Animals were closely observed for toxicity assessment, and histopathological examination was performed after the dosing period and the recovery period. Blood samples were withdrawn on day 0, 5, 7 after the first injection. 200 μL whole blood was mixed with 3 mL ACK buffer (Gibco, A10492-01) and lysed on ice for 12 min. Single-cell suspensions were prepared and further processed for surface markers staining, fixing, membrane permeabilization, and intracellular staining, according to manufacturer’s instructions. FACScelesta (BD) was used for the detection of CD3^+^CD4^+^CD25^high^ Foxp3^+^ Treg cells, CD3^+^CD4^+^ T cells, CD3^+^CD8^+^ T cells and CD3^-^CD16^+^ natural killer cells. Antibodies and reagents are listed in Supplementary Table 2.

### Statistics and reproducibility

Statistical analysis was performed using GraphPad Prism software. As indicated in the figure legends, the data were analyzed by one-way analysis of variance (ANOVA) with Dunnett’s or Tukey’s multiple comparisons. Differences in means were considered significant when the *P* value was less than 0.05. Sample size was chosen empirically according to previous studies. The number of experimental replicates was indicated in the figure legends. Animals were randomly assigned into different treatment groups and the numbers for each group were provided in the figure legends. We have complied with all relevant ethical regulations for animal use from Institutional Animal Care and Use Committee (IACUC). Investigators were not blinded. No data outliers were excluded.

### Reporting summary

Further information on research design is available in the Nature Portfolio Reporting Summary linked to this article.

### Supplementary information

Supplementary Information

Description of Additional Supplementary Files

Supplementary Data 1

Reporting Summary

## Data Availability

All data supporting the findings of this study are available within the paper and its Supplementary Information. Numerical source data for all plots and graphs can be found in Supplementary Data 1.

## References

[1] Suzuki H, Duncan GS, Takimoto H, Mak TW (1997). Abnormal development of intestinal intraepithelial lymphocytes and peripheral natural killer cells in mice lacking the IL-2 receptor β chain. J. Exp. Med..

[2] Sadlack B (1993). Ulcerative colitis-like disease in mice with a disrupted interleukin-2 gene. Cell.

[3] Willerford DM (1995). Interleukin-2 receptor α chain regulates the size and content of the peripheral lymphoid compartment. Immunity.

[4] Shevach EM (2012). Application of IL-2 therapy to target T regulatory cell function. Trends Immunol..

[5] Cheng G, Yu A, Malek TR (2011). T-cell tolerance and the multi-functional role of IL-2R signaling in T-regulatory cells. Immunol. Rev..

[6] Klatzmann D, Abbas AK (2015). The promise of low-dose interleukin-2 therapy for autoimmune and inflammatory diseases. Nat. Rev. Immunol..

[7] Saadoun D (2011). Regulatory T-cell responses to low-dose interleukin-2 in HCV-induced vasculitis. N. Engl. J. Med..

[8] Koreth J (2011). Interleukin-2 and regulatory T cells in graft-versus-host disease. N. Engl. J. Med..

[9] Boyman O, Kovar M, Rubinstein MP, Surh CD, Sprent J (2006). Selective stimulation of T cell subsets with antibody-cytokine immune complexes. Science.

[10] Spangler JB (2015). Antibodies to interleukin-2 elicit selective T cell subset potentiation through distinct conformational mechanisms. Immunity.

[11] Létourneau S (2010). IL-2/anti-IL-2 antibody complexes show strong biological activity by avoiding interaction with IL-2 receptor alpha subunit CD25. Proc. Natl. Acad. Sci. USA..

[12] Tang Q (2008). Central role of defective interleukin-2 production in the triggering of islet autoimmune destruction. Immunity.

[13] Liu R (2010). Expansion of regulatory T cells via IL-2/anti-IL-2 mAb complexes suppresses experimental myasthenia. Eur. J. Immunol..

[14] Grinberg-Bleyer Y (2010). IL-2 reverses established type 1 diabetes in NOD mice by a local effect on pancreatic regulatory T cells. J. Exp. Med..

[15] Webster KE (2009). In vivo expansion of T reg cells with IL-2-mAb complexes: induction of resistance to EAE and long-term acceptance of islet allografts without immunosuppression. J. Exp. Med..

[16] Park Y (2010). Effect of in vitro expanded CD4^+^CD25^+^Foxp3^+^ regulatory T cell therapy combined with lymphodepletion in murine skin allotransplantation. Clin. Immunol..

[17] Trotta E (2018). A human anti-IL-2 antibody that potentiates regulatory T cells by a structure-based mechanism. Nat. Med..

[18] Karakus U (2020). Receptor-gated IL-2 delivery by an anti-human IL-2 antibody activates regulatory T cells in three different species. Sci. Transl. Med..

[19] Spangler JB (2018). Engineering a single-agent cytokine/antibody fusion that selectively expands regulatory T cells for autoimmune disease therapy. J. Immunol..

[20] Yu A (2015). Selective IL-2 responsiveness of regulatory T cells through multiple intrinsic mechanisms supports the use of low-dose IL-2 therapy in type 1 diabetes. Diabetes.

[21] Lotze MT, Frana LW, Sharrow SO, Robb RJ, Rosenberg SA (1985). In vivo administration of purified human interleukin 2. I. Half-life and immunologic effects of the Jurkat cell line-derived interleukin 2. J. Immunol..

[22] Peterson LB (2018). A long-lived IL-2 mutein that selectively activates and expands regulatory T cells as a therapy for autoimmune disease. J. Autoimmun..

[23] Glassman CR (2021). Calibration of cell-intrinsic interleukin-2 response thresholds guides design of a regulatory T cell biased agonist. Elife.

[24] Ng CT (2015). Blockade of interferon beta, but not interferon alpha, signaling controls persistent viral infection. Cell Host Microbe..

[25] Ng CT, Mendoza JL, Garcia KC, Oldstone MB (2016). Alpha and beta type 1 interferon signaling: passage for diverse biologic outcomes. Cell.

[26] VanDyke D (2022). Engineered human cytokine/antibody fusion proteins expand regulatory T cells and confer autoimmune disease protection. Cell Rep..

[27] Ward NC (2018). IL-2/CD25: a long-acting fusion protein that promotes immune tolerance by selectively targeting the IL-2 receptor on regulatory T cells. J. Immunol..

[28] Humrich JY (2019). Low-dose interleukin-2 therapy in refractory systemic lupus erythematosus: an investigator-initiated, single-centre phase 1 and 2a clinical trial. Lancet Rheumatol..

[29] Yamada Y (2023). Biased IL-2 signals induce Foxp3-rich pulmonary lymphoid structures and facilitate long-term lung allograft acceptance in mice. Nat. Commun..

[30] Kim KS (2016). Dietary antigens limit mucosal immunity by inducing regulatory T cells in the small intestine. Science.

[31] Kaminski A (2023). Resident regulatory T cells reflect the immune history of individual lymph nodes. Sci. Immunol..

[32] Tao X, Fan F, Hoffmann V, Longo NS, Lipsky PE (2006). Therapeutic impact of the ethyl acetate extract of Tripterygium wilfordii Hook F on nephritis in NZB/W F1 mice. Arthritis Res. Ther..

