# Supplementary material for: A single-agent fusion of human IL-2 and anti-IL-2 antibody that selectively expands regulatory T cells
Source: Commun Biol. 2024 Mar 9;7:299. doi: 10.1038/s42003-024-05987-z (PMC10925001; doi:10.1038/s42003-024-05987-z)
Supplement: Supplementary file 1 — Supplementary Information [file 42003_2024_5987_MOESM1_ESM.pdf]

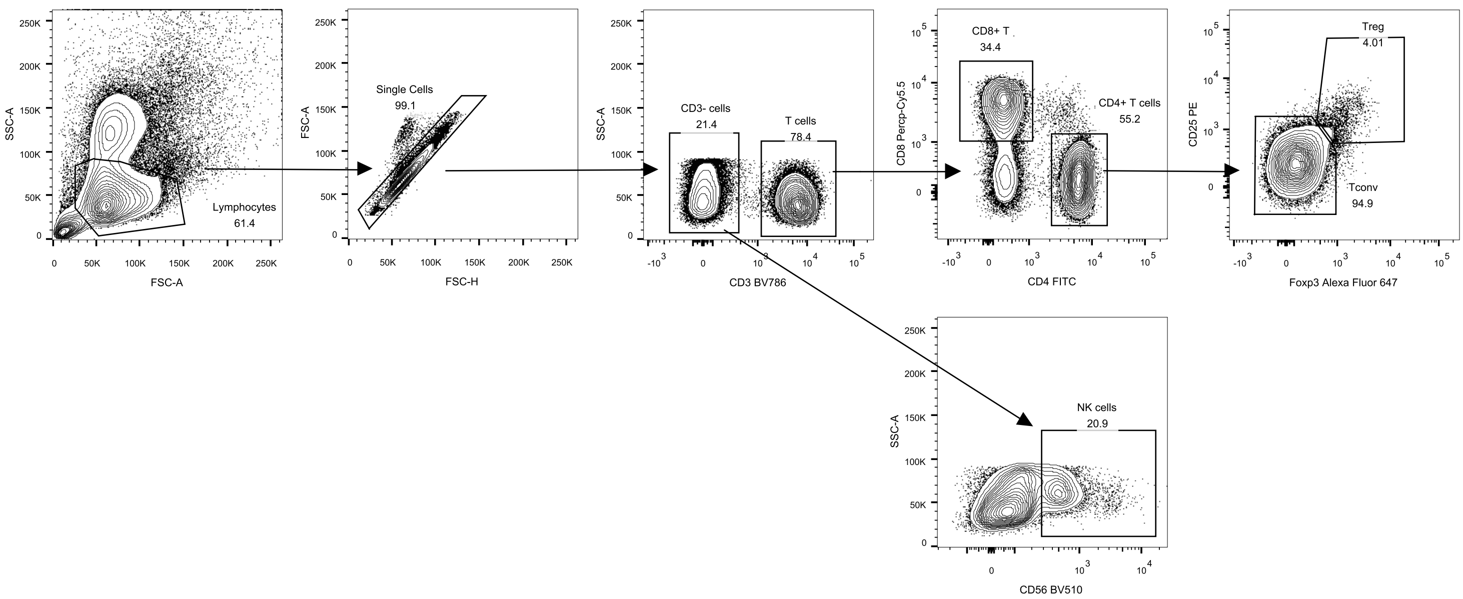

Supplementary Figure 1 | Representative flow plots of gating strategy for Treg, CD8⁺ T and NK cells in human PBMC.

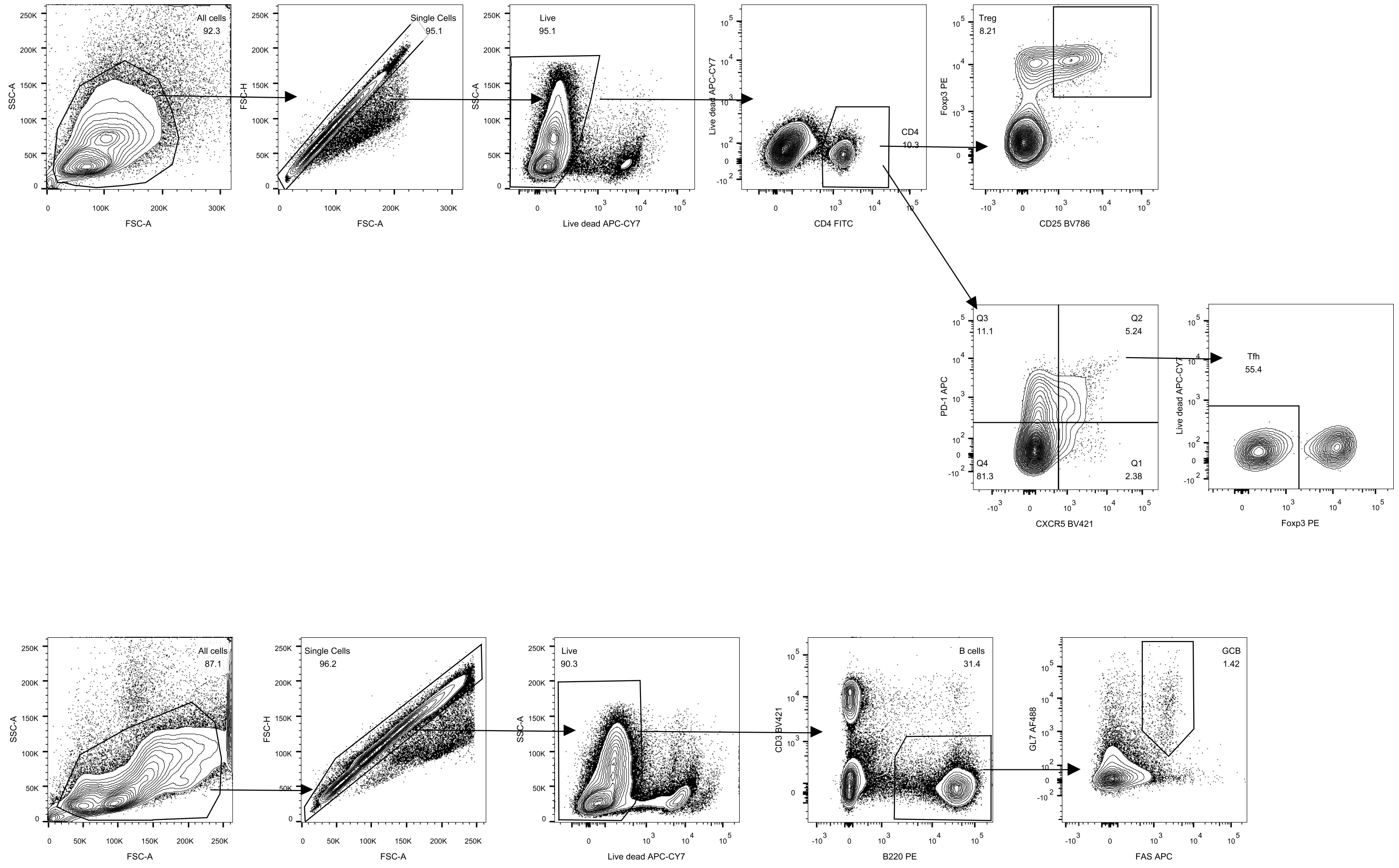

Supplementary Figure 2 | Representative flow plots of gating strategy for Treg, Tfh and GCB cells in OVA-immunized mice.

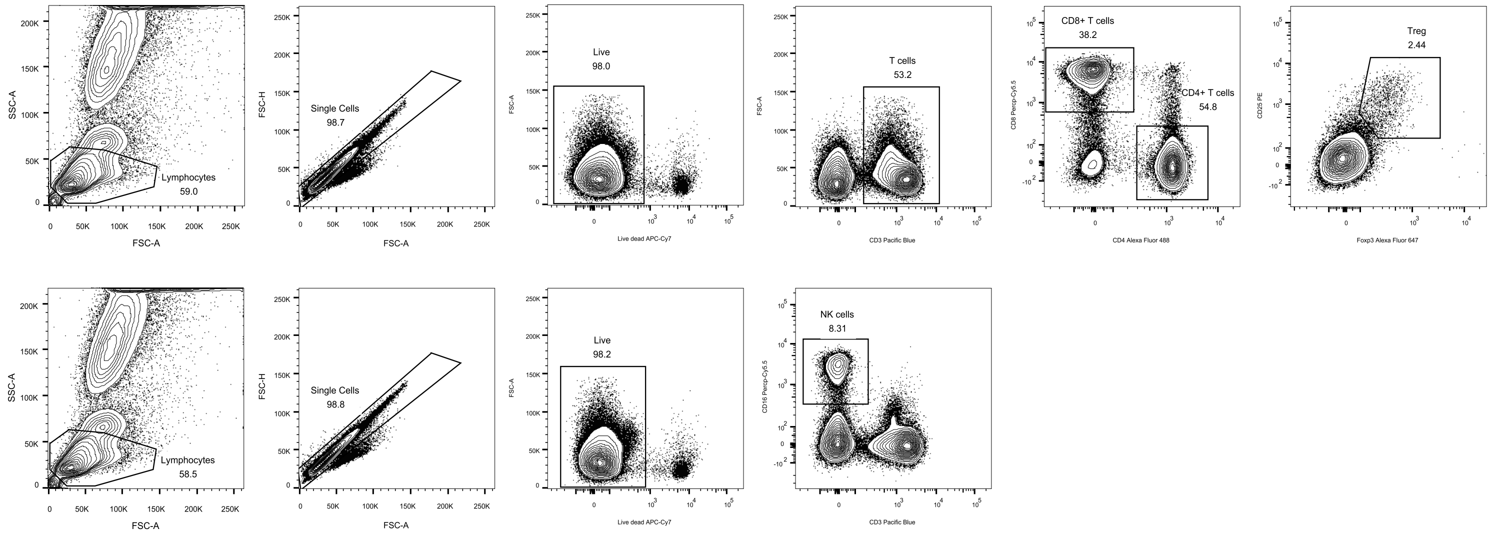

Supplementary Figure 3 | Representative flow plots of gating strategy for Treg, CD4^{+} T, CD8^{+} T and NK cells in cynomolgus monkeys.

a

Items	IL-2/SD-01clcx group (mg/kg)	Observations/Findings (Incidence)
Clinical observations		
	0.15	No abnormality
	0.03	No abnormality
Body weight		
	0.15	No abnormality
	0.03	No abnormality
Food consumption		
	0.15	No abnormality
	0.03	No abnormality
Body temperature		
	0.15	No abnormality
	0.03	No abnormality
Respiration		
	0.15	No abnormality
	0.03	No abnormality
Electrocardiogram		
	0.15	No abnormality
	0.03	No abnormality
Blood pressure		
	0.15	No abnormality
	0.03	No abnormality
Ophthalmic investigations		
	0.15	No abnormality
	0.03	No abnormality
Hematology		
	0.15	No abnormality
	0.03	No abnormality
Coagulation panels		
	0.15	No abnormality
	0.03	No abnormality
Blood biochemical items		
	0.15	No abnormality
	0.03	No abnormality
Immunotoxicity (complement, CRC, CRP, lgG, lgA, lgM, Cytokine)		
	0.15	No abnormality
	0.03	No abnormality
Bone marrow cytology		
	0.15	No abnormality
	0.03	No abnormality
Urinalysis		
	0.15	No abnormality
	0.03	No abnormality
Anatomical observation		
	0.15	No abnormality
	0.03	No abnormality
Organ weights		
	0.15	No abnormality
	0.03	No abnormality
Subcutaneous injection sites		
	0.15	No abnormality
	0.03	No abnormality
Histopathological examination		
	0.15	Minimal myometrial/endometrial multifocal perivascular mononuclear cell infiltration (1/3), and disappered at the end of the recovery period.
	0.03	Minimal myometrial/endometrial multifocal perivascular mononuclear cell infiltration (1/3), and disappered at the end of the recovery period.

b

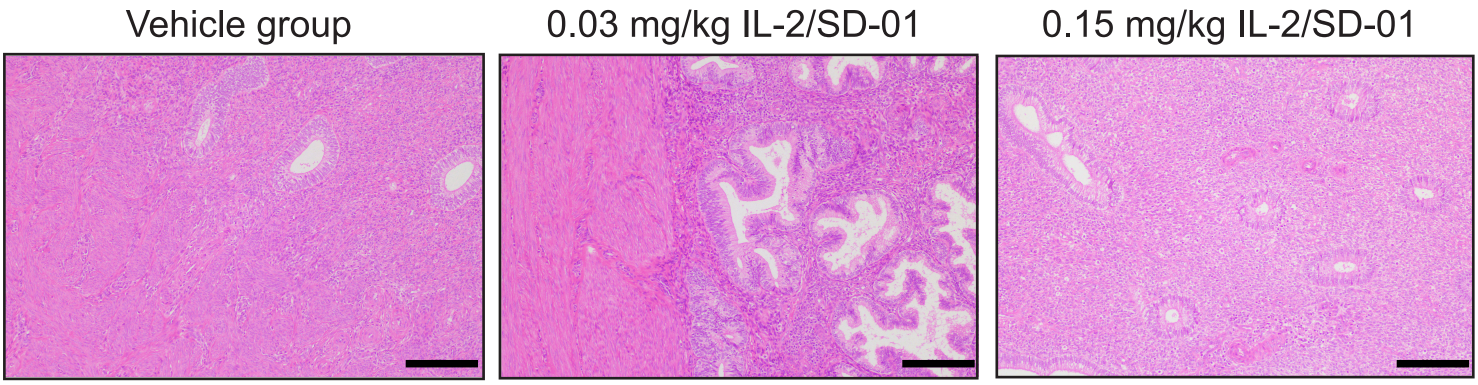

Supplementary Figure 4 | IL-2/SD-01 has a favorable safety profile in nonhuman primates after 13-week treatment. (**a**) No mortality and major drug-related adverse effects were observed in any group, except that the minimal myometrial/endometrial multifocal perivascular mononuclear cell infiltration was observed microscopically in one femal of each IL-2/SD-01 group at the end of the 13-week treatment (1/3 per group), which was recovered at the end of the recovery period (2/2 per group). (**b**) Minimal myometrial/endometrial multifocal perivascular mononuclear cell infiltration was observed in IL-2/SD-01-treating groups (H&E staining). Scale bar, 200μm.

Supplementary Table 1 | Antibodies and reagents used for human PBMC STAT5 phosphorylation assay.

Antibody/Reagent	Company	Catalog number	Dilution factor
Human CD3, BV786, UCHT1	BD Biosciences	565491	1:100
Human CD8, PerCP-Cy5.5, SK1	BD Biosciences	565310	1:100
Human CD4, FITC, RPA-T4	BD Biosciences	555346	1:100
Human CD25, PE, M-A251	BD Biosciences	555432	1:40
pStat5 (pY694), BV421, 47	BD Biosciences	562984	1:40
Human FoxP3, AF647	BioLegend	320014	1:30
Human CD56, BV510, MY31	BD Biosciences	742658	1:50

Supplementary Table 2 | Antibodies and reagents used for whole blood STAT5 phosphorylation assay and pharmacodynamics study in cynomolgus monkey.

Antibody/Reagent	Company	Catalog number	Dilution factor
ACK lysing buffer	Gibco	A10492-01	
Fixable Viability Stain 780	eBioscience	65-0865-18	1:1000
Human BD Fc Block	BD Biosciences	564219	1:200
Human CD3, Pacific Blue, SP34-2	BD Biosciences	558124	1:100
Human CD4, AF488, OKT4	BioLegend	317420	1:100
Human CD8, PerCP-Cy5.5, SK1	BD Biosciences	565310	1:100
pStat5 (pY694), BV421, 47	BD Biosciences	562984	1:40
Human CD25, PE	BioLegend	302606	1:40
Human FoxP3, AF647	BioLegend	320014	1:30
Human CD16, PerCP-Cy5.5, 3G8	BD Biosciences	560717	1:50
Human Ki67, BV786, B56	BD Biosciences	563756	1:50
Foxp3/Transcription Factor Staining Buffer Set	eBioscience	00-5523-00	

Supplementary Table 3 | Antibodies and reagents used for pharmacodynamics study in mice.

Antibody/Reagent	Company	Catalog number	Dilution factor
Mouse CD3, APC-Cy7, 145-2C11	BioLegend	100329	1:100
Mouse CD8a, PE, 53-6.7	BioLegend	100708	1:100
Mouse CD4, PE-Cy7, RM4-5	eBioscience	25-0042-82	1:100
Mouse CD25, PerCP-Cy5.5, PC61	BD Biosciences	561112	1:40
Mouse Foxp3, AF488, MF-14	BioLegend	126405	1:30
Mouse Ki67, PE-Cy7, SolA15	eBioscience	25-5698-82	1:50
True-Nuclear^{TM} Transcription Factor Buffer Set	BioLegend	424401	

Supplementary Table 4 | Antibodies and reagents used for inhibition of humoral immunity in mice immunized with OVA.

Antibody/Reagent	Company	Catalog number	Dilution factor
CD4, FITC, GK1.5	Thermo Fisher	100329	1:100
CD25, APC, PC61.5	Thermo Fisher	100708	1:40
Foxp3, BV421, MF23	BD Biosciences	562996	1:30
CXCR5, PE, SPRCL5	eBioscience	12-7185-82	1:50
PD-1, PE-Cy7, 29F.1A12	BioLegend	135216	1:50
Fas, PE, 15A7	eBioscience	12-0951-81	1:50
GL-7, AF488, GL-7	eBioscience	53-5902-82	1:50
B220, APC, RA3-6B2	Thermo Fisher	17-0452-82	1:50
