# Supplementary material for: A single-agent fusion of human IL-2 and anti-IL-2 antibody that selectively expands regulatory T cells
Source: Commun Biol. 2024 Mar 9;7:299. doi: 10.1038/s42003-024-05987-z (PMC10925001; doi:10.1038/s42003-024-05987-z)
Supplement: Supplementary file 4 — Reporting Summary [file 42003_2024_5987_MOESM4_ESM.pdf]

nature portfolio

Corresponding author(s): Cheng Liao, COMMSBIO-23-1531B

Last updated by author(s): Feb 7, 2024



# Reporting Summary

Nature Portfolio wishes to improve the reproducibility of the work that we publish. This form provides structure for consistency and transparency in reporting. For further information on Nature Portfolio policies, see our Editorial Policies and the Editorial Policy Checklist.

## Statistics

For all statistical analyses, confirm that the following items are present in the figure legend, table legend, main text, or Methods section.

| n/a | Confirmed | |
|---|---|---|
| ☐ | ☒ | The exact sample size (*n*) for each experimental group/condition, given as a discrete number and unit of measurement |
| ☐ | ☒ | A statement on whether measurements were taken from distinct samples or whether the same sample was measured repeatedly |
| ☐ | ☒ | The statistical test(s) used AND whether they are one- or two-sided<br>*Only common tests should be described solely by name; describe more complex techniques in the Methods section.* |
| ☒ | ☐ | A description of all covariates tested |
| ☐ | ☒ | A description of any assumptions or corrections, such as tests of normality and adjustment for multiple comparisons |
| ☐ | ☒ | A full description of the statistical parameters including central tendency (e.g. means) or other basic estimates (e.g. regression coefficient) AND variation (e.g. standard deviation) or associated estimates of uncertainty (e.g. confidence intervals) |
| ☐ | ☒ | For null hypothesis testing, the test statistic (e.g. *F*, *t*, *r*) with confidence intervals, effect sizes, degrees of freedom and *P* value noted<br>*Give P values as exact values whenever suitable.* |
| ☒ | ☐ | For Bayesian analysis, information on the choice of priors and Markov chain Monte Carlo settings |
| ☒ | ☐ | For hierarchical and complex designs, identification of the appropriate level for tests and full reporting of outcomes |
| ☒ | ☐ | Estimates of effect sizes (e.g. Cohen's *d*, Pearson's *r*), indicating how they were calculated |

*Our web collection on statistics for biologists contains articles on many of the points above.*

## Software and code

Policy information about availability of computer code

| | |
|---|---|
| Data collection | FACSDiva (BD Biosciences), Biacore 8K (GE Healthcare) 3D HISTECH scanner |
| Data analysis | HALO (v3.3.2541.301) FlowJo 10.9 (BD Biosciences), Graphpad Prism 9.3.1, Biacore 8K Control and Evaluation Software |

For manuscripts utilizing custom algorithms or software that are central to the research but not yet described in published literature, software must be made available to editors and reviewers. We strongly encourage code deposition in a community repository (e.g. GitHub). See the Nature Portfolio guidelines for submitting code & software for further information.

## Data

Policy information about availability of data

All manuscripts must include a data availability statement. This statement should provide the following information, where applicable:

- Accession codes, unique identifiers, or web links for publicly available datasets
- A description of any restrictions on data availability
- For clinical datasets or third party data, please ensure that the statement adheres to our policy

All data associated with this study are present in the manuscript. There is no data with mandated deposition.

## Human research participants

Policy information about studies involving human research participants and Sex and Gender in Research.

| | |
|---|---|
| Reporting on sex and gender | The information was not collected. |
| Population characteristics | Healthy donors |
| Recruitment | Human peripheral blood mononuclear cells were commercially purchased from SailyBio. |
| Ethics oversight | Approval of the Ethics Committee of Shanghai Tenth People's Hospital (China) |

Note that full information on the approval of the study protocol must also be provided in the manuscript.

# Field-specific reporting

Please select the one below that is the best fit for your research. If you are not sure, read the appropriate sections before making your selection.

☒ Life sciences ☐ Behavioural & social sciences ☐ Ecological, evolutionary & environmental sciences

For a reference copy of the document with all sections, see nature.com/documents/nr-reporting-summary-flat.pdf

# Life sciences study design

All studies must disclose on these points even when the disclosure is negative.

| | |
|---|---|
| Sample size | No statistical methods were used to predetermine sample size. Sample size was chosen empirically according to previous studies, balancing statistical robustness and animal welfare. |
| Data exclusions | No data were excluded from the analysis |
| Replication | The number of experimental replicates was indicated in the figure legends |
| Randomization | Animals were randomly assigned into different treatment groups |
| Blinding | Investigators were non blinded, but for in-vitro and ex-vivo studies, the data were reproduced by different technical personnels and for in-vivo studies, the personnels involved in the group allocation, treating animals and data collection are unaware of the hypothesis or expected outcome. |

# Reporting for specific materials, systems and methods

We require information from authors about some types of materials, experimental systems and methods used in many studies. Here, indicate whether each material, system or method listed is relevant to your study. If you are not sure if a list item applies to your research, read the appropriate section before selecting a response.

### Materials & experimental systems

| n/a | Involved in the study | |
|---|---|---|
| ☐ | ☒ | Antibodies |
| ☒ | ☐ | Eukaryotic cell lines |
| ☒ | ☐ | Palaeontology and archaeology |
| ☐ | ☒ | Animals and other organisms |
| ☒ | ☐ | Clinical data |
| ☒ | ☐ | Dual use research of concern |

### Methods

| n/a | Involved in the study | |
|---|---|---|
| ☒ | ☐ | ChIP-seq |
| ☐ | ☒ | Flow cytometry |
| ☒ | ☐ | MRI-based neuroimaging |

## Antibodies

| | |
|---|---|
| Antibodies used | Antibodies and reagents used for human PBMC STAT5 phosphorylation assay<br>Marker, dye, clone, company, catalog number<br>Human CD3, BV786, UCHT1, BD Biosciences, 565491<br>Human CD8, PerCP-Cy5.5, SK1, BD Biosciences, 565310<br>Human CD4, FITC, RPA-T4, BD Biosciences, 555346 |

Human CD25, PE, M-A251, BD Biosciences, 555432
pStat5 (pY694), BV421, 47, BD Biosciences, 562984
Human FoxP3, AF647, BioLegend, 320014
Human CD56, BV510, MY31, BD Biosciences, 742658

Antibodies used for whole blood STAT5 phosphorylation assay and pharmacodynamics study in cynomolgus monkey
Marker, dye, clone, company, catalog number
Human CD3, Pacific Blue, SP34-2, BD Biosciences, 558124
Human CD4, AF488, OKT4, BioLegend, 317420
Human CD8, PerCP-Cy5.5, SK1, BD Biosciences, 565310
pStat5 (pY694), BV421, 47, BD Biosciences, 562984
Human CD25, PE, BioLegend, 302606
Human FoxP3, AF647, BioLegend, 320014
Human CD16, PerCP-Cy5.5, 3G8, BD Biosciences, 560717
Human Ki67, BV786, B56, BD Biosciences, 563756

Antibodies and reagents used for pharmacodynamics study in mice
Marker, dye, clone, company, catalog number
Mouse CD3, APC-Cy7, 145-2C1,BioLegend, 100329
Mouse CD8a, PE, 53-6, BioLegend, 100708
Mouse CD4, PE-Cy7, RM4, eBioscience, 25-0042-82
Mouse CD25, PerCP-Cy5.5, PC61, BD Biosciences, 561112
Mouse Foxp3, AF488, MF-1, BioLegend, 126405
Mouse Ki67, PE-Cy7, SolA1, eBioscience 25-5698-82

Antibodies and reagents used for inhibition of humoral immunity in mice immunized with OVA
Marker, dye, clone, company, catalog number
CD4, FITC, GK1.5, Thermo Fisher, 100329
CD25, APC, PC61.5, Thermo Fisher, 100708
Foxp3, BV421, MF23, BD Biosciences, 562996
CXCR5, PE, SPRCL5, eBioscience, 12-7185-82
PD-1, PE-Cy7, 29F.1A12, BioLegend, 135216
Fas, PE, 15A7, eBioscience, 12-0951-81
GL-7, AF488, GL-7 eBioscience 53-5902-82
B220, APC, RA3-6B2, Thermo Fisher, 17-0452-82

Validation

The FACS antibodies have been titrated on human PBMC or mouse splenocytes for meaningful biological patterns by monitoring the frequencies of positive cells known from previous experience or the available literature. Detailed information and the manufactures' recommendations can be found in https://www.bdbiosciences.com, https://www.biolegend.com and https://www.thermofisher.cn/cn/zh/home/life-science/antibodies/ebioscience.html

## Animals and other research organisms

Policy information about studies involving animals; ARRIVE guidelines recommended for reporting animal research, and Sex and Gender in Research

Laboratory animals

For pharmacokinetics study in mice, wild type BALB/c mice at the age of 6-8 weeks were purchased from Jihui Laboratory Animal Co. Ltd. For pharmacodynamics study in mice, wild type BALB/c mice at the age of 6-8 weeks were purchased from Beijing Vital River Laboratory Animal Technology Co. Ltd. For DTH study, ICR mice at the age of 6 weeks were purchased from Shanghai Lab of Animal Research Center. For OVA study in mice, C56B16/J mice at the age of 6-8 weeks were purchased from Shanghai Lab of Animal Research Center. For DSS-induced UC model of rat, male Sprague-Dawley rats were provided by the Beijing Vital River Laboratory Animal Technology Co., Ltd. For mice model of SLE, BALB/c and MRL/lpr mice were purchased from Shanghai SLAC Laboratory Animal Co., Ltd. For pharmacokinetics and pharmacodynamics studies in cynomolgus monkeys, monkeys were purchased from InnoStar Bio-tech Nantong Co. Ltd.

Wild animals

The study did not involve wild animals

Reporting on sex

For pharmacokinetics and pharmacodynamics studies in mice, female mice were used. For DTH and OVA study, male mice were used. For DSS-induced UC model of rat, male rats were used. For mice model of SLE, female mice were used. For pharmacokinetics and pharmacodynamics studies in cynomolgus monkeys, both female and male monkeys were used.

Field-collected samples

No field collection was performed

Ethics oversight

All animal-related procedures were in compliance with relevant ethical regulations for animal usage from Institutional Animal Care and Use Committee (IACUC)

Note that full information on the approval of the study protocol must also be provided in the manuscript.

# Flow Cytometry

## Plots

Confirm that:

☒ The axis labels state the marker and fluorochrome used (e.g. CD4-FITC).

☒ The axis scales are clearly visible. Include numbers along axes only for bottom left plot of group (a 'group' is an analysis of identical markers).

☒ All plots are contour plots with outliers or pseudocolor plots.

☒ A numerical value for number of cells or percentage (with statistics) is provided.

## Methodology

Sample preparation

For in vitro human PBMC pSTAT5 assay, 1x10^6 PBMC was fixed with Cytofix Fixation buffer (BD Biosciences), followed by permeabilization with Phosflow Perm Buffer III (BD Biosciences) at -20 degree overnight. Then cells were blocked with Human BD Fc Block (BD Biosciences) on ice for 15 minutes. Extracellular and intracellular staining were performed simutaneously on ice for 60 minutes. For cynomolgus monkey pSTAT5 assay, whole blood was fixed with 1 x BD PhosFlow Lyse/Fix buffer at 37 degree for 10 minutes and the following procedures were in accordance with human PBMC pSTAT5 assay.

For experiments in OVA-immunized mice, the spleen cells were divided into two parts. For Treg staining, 1x10^6 mouse splenocytes per 50µL were blocked with BD Fc Block, then stained live/dead dye for 15 min at room temperature. Extracellular staining was performed in FACS buffer for 30 min on ice followed by fixation using FoxP3 buffer kit (eBioscience) for 30 min at room temperature. Intracellular staining was performed in permeabilization buffer provided in the FoxP3 buffer kit for 30 min at room temperature. Then stained with Foxp3 PE mAb for 30 min at room temperature. For GCB staining, 1x10^6 mouse splenocytes per 50µL were blocked with BD Fc Block, then stained live/dead dye for 15 min at room temperature. Extracellular staining was performed in FACS buffer for 30 min on ice.

Instrument

All data acquisitions were performed on BD Celasta.

Software

All flow cytometry data was collected using FACSDiva (BD Biosciences) and analyzed using FlowJo 10.9 (BD Biosciences).

Cell population abundance

Sorted populations were checked for purity post-sort via flow cytometry and were >95% pure.

Gating strategy

For in vitro human PBMC pSTAT5 assay, cells were gated on FSC/SSC according to cell size and granularity. Regulatory T cells were gated as CD3+CD4+CD25+FoxP3+, CD8+ T cells were gated as CD3+CD8+ and NK cells were gated as CD3-CD56+.

For experiments in OVA-immunized mice, ells were gated on FSC/SSC according to cell size and granularity, and dead cells were gated out. Regulatory T cells were gated as CD4+CD25+FoxP3+, Tfh cells were gated as CD4+PD-1 high CXCR5 high Foxp3-, and GCB cells were gated as B220+GL7+Fas+.

For cynomolgus monkey pSTAT5 assay, cells were gated on FSC/SSC according to cell size and granularity, and dead cells were gated out. Regulatory T cells were gated as CD3+CD4+CD25+FoxP3+, CD4+ T cells were gated as CD3+CD4+, CD8+ T cells were gated as CD3+CD8+ and NK cells were gated as CD3-CD16+.

☒ Tick this box to confirm that a figure exemplifying the gating strategy is provided in the Supplementary Information.
